# NADP^+^ is an endogenous PARP inhibitor in DNA damage response and tumor suppression

**DOI:** 10.1038/s41467-019-08530-5

**Published:** 2019-02-11

**Authors:** Chunjing Bian, Chao Zhang, Tao Luo, Aditi Vyas, Shih-Hsun Chen, Chao Liu, Muzaffer Ahmad Kassab, Ying Yang, Mei Kong, Xiaochun Yu

**Affiliations:** 1 Department of Cancer Genetics and Epigenetics, Beckman Research Institute, City of Hope Medical Center, Duarte, CA 91010 USA; 2 Department of General Surgery of Xuanwu Hospital, Capital Medical University, 100053 Beijing, China; 3 Vascular Surgery Department of Xuanwu Hospital, Institute of Vascular Surgery, Capital Medical University, 100053 Beijing, China; 4 Department of Cancer Biology, Beckman Research Institute, City of Hope Medical Center, Duarte, CA 91010 USA

## Abstract

ADP-ribosylation is a unique posttranslational modification catalyzed by poly(ADP-ribose) polymerases (PARPs) using NAD^+^ as ADP-ribose donor. PARPs play an indispensable role in DNA damage repair and small molecule PARP inhibitors have emerged as potent anticancer drugs. However, to date, PARP inhibitor treatment has been restricted to patients with BRCA1/2 mutation-associated breast and ovarian cancer. One of the major challenges to extend the therapeutic potential of PARP inhibitors to other cancer types is the absence of predictive biomarkers. Here, we show that ovarian cancer cells with higher level of NADP^+^, an NAD^+^ derivative, are more sensitive to PARP inhibitors. We demonstrate that NADP^+^ acts as a negative regulator and suppresses ADP-ribosylation both in vitro and in vivo. NADP^+^ impairs ADP-ribosylation-dependent DNA damage repair and sensitizes tumor cell to chemically synthesized PARP inhibitors. Taken together, our study identifies NADP^+^ as an endogenous PARP inhibitor that may have implications in cancer treatment.

## Abstract (web summary)

Cancer cells respond differently to inhibitors of Poly (ADP-ribose) polymerase. Here the authors reveal that ovarian cancer cells with higher cellular NADP+ levels are more sensitive to clinically relevant PARP1 inhibitors and show that NADP+ act as an endogenous inhibitor of PARP enzymes.

## Introduction

ADP-ribosylation is a unique posttranslational modification synthesized in response to genotoxic stress that acts as the earliest alarm for sensing DNA damage response^1^. ADP-ribosylation is catalyzed by a group of poly(ADP-ribose) polymerases (PARPs), which is a protein family comprising 17 members^2,3^. Using NAD^+^ as the ADP-ribose (ADPr) donor, PARPs transfer ADPr moiety onto the side chains of arginine, aspartic acid, glutamic acid, cysteine, lysine, serine, and tyrosine residues of target proteins^4–12^. After transferring the first ADPr onto the target proteins, other ADPrs can be sequentially added onto the first ADPr with 1'–2' glycosidic bond between ribose units and continuous polymerization leads to the formation of both linear and branched polymer chains of ADPr^13^.

To date, several PARPs have been reported to participate in DNA damage response^1,14,15^. Among these PARPs, PARP1 is the most potent enzyme to catalyze poly(ADP-ribosyl)ation (PARylation) accounting for 80–90% of DNA damage-induced PARylation^1,16,17^. Besides PARP1, PARP2 is also involved in DNA damage-induced PARylation^18,19^. Notably, mice with genetic disruption of *Parp1* gene are viable and do not show obvious developmental defects. However, disruption of both *Parp1* and *Parp2* in mice impairs gastrulation and causes early embryonic lethality^20^, demonstrating that these two PARPs may have redundant functions. Moreover, PARP3 and PARP10 have been shown to participate in DNA damage repair^21–23^, with PARP10 catalyzing mono(ADP-ribosyl)ation (MARylation) on its target substrates^24^. Although NAD^+^-binding pockets are quite similar in these enzymes; however, contrary to PARP1 and PARP2, PARP10 lacks the key residue required for polymerization of ADPr, which could likely account for its lack of PARylation potential^24,25^.

In response to DNA damage, PARPs consume up to 90% of cellular NAD^+^ to catalyze massive ADP-ribosylation at the sites of DNA lesions in a very short period of time^26^. To date, numerous ADP-ribosylation substrates have been identified using unbiased proteomic screenings^6,9,27^. Since each ADPr contains two phosphate moieties, ADP-ribosylation brings huge amount of negative charges to DNA lesions. The negative charge is likely to promote relaxation of higher-order of chromatin structure due to the charge repulsion of the negatively charged phosphates in the genomic DNA backbone^28^. In addition, over the past 15 years, several ADPr-binding modules have been identified, suggesting that ADP-ribosylation functions as a signaling moiety to mediate the recruitment of DNA damage repair factors^29^. We and others have characterized several PARylation readers in DNA damage repair factors and chromatin remodeling complexes^11,29^. Thus ADP-ribosylation plays an important role in DNA damage repair.

Regulation of PARylation process has been studied over the past few decades. One of the most important pathways in PARylation is the NAD^+^ biogenesis. Although de novo generation of NAD^+^ is a very complicated process that may be associated with several pathways and >80 enzymes, NAD^+^ can be recycled following PARylation^30^. In nucleus, nicotinamide (NAM), the by-product of PARylation, is converted into nicotinamide mono-nucleotide (NMN) via phosphorylation by nicotinamide phosphoribosyltransferase (NAMPT)^31^. NMN is covalently linked to an AMP moiety from an ATP, and this reaction is catalyzed by nicotinamide mono-nucleotide adenylyl transferase1 (NMNAT1)^32^. Thus the rate limiting steps to generate NAD^+^ in nucleus are controlled by NAMPT and NMNAT1^31,32^. Moreover, NAD^+^ can be phosphorylated to NADP^+^ by NAD kinase (NADK)^33^. Thus these enzymes together may change the levels of NAD^+^ and regulate PARylation. In particular, recent evidence suggests that NMNAT1 promotes PARP1’s activity during adipogenesis^34^.

Although oncogenic mutations of PARPs have not been identified, PARP inhibitors have been successfully utilized in cancer chemotherapy^35,36^. Current PARP inhibitors are designed to compete with NAD^+^ for occupying the catalytic cages of PARPs, especially those present in PARP1 and PARP2. These inhibitors trap PARP1 and PARP2 at DNA lesions and abolish PARylation-mediated biological processes, such as DNA damage repair^37,38^. Accumulated evidence has also suggested that tumor cells with impaired homologous recombination (HR) repair are hypersensitive to PARP inhibitors^39^. Since BRCA1 and BRCA2 play indispensable roles in HR repair^40^, PARP inhibitor treatment specifically kills tumor cells containing mutations in *BRCA1* and *BRCA2* genes^41,42^. Over the past few years, US Food and Drug Administration (FDA) approved three types of PARP inhibitors including olaparib, rucaparib, and niraparib to treat breast, ovarian, and prostate cancers with BRCA1 and BRCA2 mutations. Nevertheless, recent clinical trials show that not all tumors with BRCA1/2 mutations responded efficiently to PARP inhibitor treatment^43,44^. Paradoxically, PARP inhibitors were found to be effective in treating other types of cancers lacking BRCA1/2 mutations or defects in HR pathway^45,46^. Thus, in conjugation to BRCA mutations, additional factors may be involved in the cellular sensitivity to PARP inhibitor treatment.

In order to extend the therapeutic potential of PARP inhibitors in cancer treatment, we explored predictive biomarkers for the PARP inhibitor treatment in ovarian cancers and found that NADP^+^ is an endogenous inhibitor of ADP-ribosylation. Our results suggest that tumor cells with higher levels of NADP^+^ are hypersensitive to PARP inhibitor treatment.

## Results

### Ovarian cancer cell sensitivity to PARP inhibitor

**Fig. 1:**
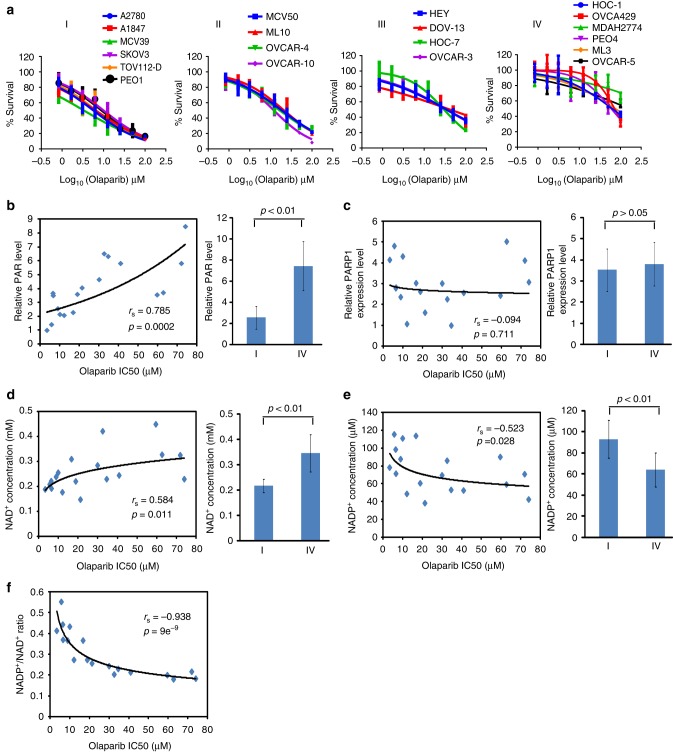
The NADP^+^/NAD^+^ ratio is associated with the cell sensitivity to poly(ADP-ribose) polymerase (PARP) inhibitor. **a** The sensitivity of 20 ovarian cancer cell lines to olaparib. Twenty ovarian cancer cell lines were treated with olaparib at the indicated doses. Cells were examined by MTT (3-[4,5-dimethylthiazol-2-yl]-2,5 diphenyl tetrazolium bromide) assays. Based on the sensitivity to olaparib, the cell were classified into four groups. The data were summarized from three independent experiments. Data are presented as mean ± SD. **b** Spearman’s correlation analysis was performed between DNA damage-induced PARylation levels and the IC50 of olaparib. DNA damage-induced endogenous PARylation levels were summarized from the western blotting in Supplementary Figure 3. IC50 of olaparib was calculated from the MTT assays. DNA damage-induced endogenous PARylation levels correlated with IC50 of olaparib (left) and PARylation levels in the olaparib-sensitive cells are markedly lower than that in the olaparib-insensitive cells (right). **c** The expression level of PARP1 is not associated with the sensitivity to olaparib. PARP1 expression levels were summarized from western blotting results in Supplementary Figure 4 by using ImageJ. No correlation was seen between PARP1 expression levels and IC50 of olaparib (left); and no difference was observed in the PARylation levels between the olaparib-sensitive cells and the olaparib-insensitive cells (right). **d**, **e** Correlation analysis of NAD^+^ and NADP^+^ concentrations and cell sensitivity to olaparib. NAD^+^ and NADP^+^ concentrations were measured in 20 ovarian cancer cell lines. Both NAD^+^ and NADP^+^ concentrations are associated with the sensitivity to olaparib (left). The NAD^+^ concentrations in the olaparib-sensitive cells are markedly higher than that in the olaparib-insensitive cells (**d**, right). And the NADP^+^ concentrations in the olaparib-sensitive cells is markedly lower than that in the olaparib-insensitive cells (**e**, right). **f** Sensitivity of ovarian cancer cells to olaparib is associated with the NADP^+^/NAD^+^ ratio. The NADP^+^/NAD^+^ ratio was calculated. NADP^+^/NAD^+^ ratio shows significant correlation with sensitivity of ovarian cancer cells to olaparib. *p* Value of Spearman’s correlation was calculated by R function, cor.test() (**b**–**e**, left; **f**). Statistical significance of the difference between the olaparib-sensitive cells and olaparib-insensitive cells was analyzed using two-tailed unpaired Student’s *t* tests (**b**–**e**, right)

To investigate the sensitivity of ovarian cancer cells to PAPR inhibitor treatment, we treated 20 ovarian cancer cell lines with olaparib, which is a potent PARP inhibitor^47^. Based on the screening results, we classified these cell lines into four groups from I to IV. Among the 20 cell lines, 6 cell lines were hypersensitive to olaparib with an IC50 < 10 μM and were placed in group I. In contrast, on the extreme side, 6 cell lines in group IV were insensitive to olaparib with an IC50 > 50 μM. The remaining eight cell lines in group II and III had intermediate sensitivity to olaparib treatment with the cell lines in group II having higher sensitivity than group III (Fig. 1a). To exclude any olaparib off-target effects, we examined the cellular sensitivity to niraparib, another potent PARP inhibitor^48,49^. We observed consistent results when compared to olaparib (Supplementary Figure 1).

Accumulated evidence suggests that tumors with BRCA1/2 mutation are hypersensitive to PARP inhibitor treatment. However, in our ovarian cancer cell lines, the efficacy of olaparib did not always corroborate with BRCA1/ 2 mutations. In group I, PEO1 harbors a *BRCA2* mutation^50^, thus represents a BRCA2-deficient cell line. Moreover, the expression levels of BRCA1 were remarkably reduced in A1847 cells due to the hypermethylation of *BRCA*1 promoter. However, at least four other cell lines in group I including A2780, MCV39, SKOV3, and TOV112-D express normal level of wild-type BRCA1 and BRCA2 and do not have obvious HR defects (Supplementary Figure 2a, e)^51,52^. In addition, we examined HR repair in groups II–IV and we did not observe any HR defect in these cancer cell lines (Supplementary Figure 2b-e). Thus additional factors apart from the status of BRCA1/2 or HR may also contribute to the sensitivity of cancer cells to PARP inhibitors.

Since PARP inhibitors specifically suppress PARylation, we wondered whether there was a difference in the endogenous levels of PARylation in these ovarian cancer cells. To examine the DNA damage-induced PARylation, we treated ovarian cancer cells with methyl methanesulfonate (MMS), an alkylating agent that induces DNA damage. The DNA damage-induced PARylation was examined using both western blotting and dot blotting assays. We found that DNA damage-induced PARylation correlated with the cellular sensitivity to olaparib, and consequently PARylation levels in the group IV cells were markedly higher than those in group I cells (Fig. 1b and Supplementary Figure 3). These results suggest that lower levels of DNA damage-induced PARylation in ovarian cancer increase their sensitivity to PARP inhibitor treatment. Since majority of DNA damage-induced PARylation is primarily mediated by PARP1, we examined PARP1 expression levels in these ovarian cancer cells. However, we did not observe any significant correlation between the expression of PARP1 and the cellular sensitivity to olaparib treatment (Fig. 1c and Supplementary Figure 4). We also examined the PARylation levels without genotoxic stress. However, we did not observe any obvious correlation between PARylation levels and cellular sensitivity to olaparib treatment (Supplementary Figure 5).

Since NAD^+^ acts as ADPr donor for PARylation, majority of the PARP inhibitors are designed to compete with NAD^+^ at the enzyme catalytic cage of the enzymes^37,38^. Accordingly, cells with lower levels of NAD^+^ may be more sensitive to PARP inhibitor than their counterparts with higher level of NAD^+^^53^. Thus, when we examined the average levels of NAD^+^, we found that the average levels of NAD^+^ in the group IV cell lines were relatively higher than that in the group I cell lines; however, a relatively weak correlation was observed between the levels of NAD^+^ and the cellular sensitivity to PARP inhibitors *r*_s_ = 0.584, *p* = 0.011 (*p* value was calculated by R function, cor.test()) (Fig. 1d and Supplementary Figure 6).

Next, we asked whether there were additional factors regulating the recognition of NAD^+^ by PARP1. Based on the structure analysis, the catalytic cage of PARP1 accommodates NAD^+^ for the chemical reaction^54^. However, NADP^+^, a derivative of NAD^+^ with 2'-hydroxide radical phosphorylated on the adenine-ribose side, can also fit into the catalytic cage of PARP1 (Supplementary Figure 7) and may regulate or participate in the PARP1-dependent PARylation. Based on these observations, we next examined the level of NADP^+^. Interestingly, the levels of NADP^+^ were apparently upregulated in the group I cells, and the NADP^+^/NAD^+^ ratio showed a strong correlation to PARP inhibitor sensitivity (Fig. 1e, f and Supplementary Figure 6). Collectively, these results suggest that ovarian cancer cells with higher NADP^+^/NAD^+^ ratio are more sensitive to PARP inhibitor treatment.

In order to understand the molecular mechanism regulating the NADP^+^/NAD^+^ ratio, we examined the biogenesis of NAD^+^ and NADP^+^. It is well established that the rate limiting step during NAD^+^ is catalyzed by NAMPT (Supplementary Figure 8a)^31^. Thus we examined and found a positive correlation between the levels of NAD^+^ and the expression levels of NAMPT (Supplementary Figure 8b). Moreover, the conversion of NAD^+^ to NADP^+^ is catalyzed by a solo enzyme, NADK (Supplementary Figure 8a)^33^, which adds a phospho-group at 2' position of adenine-ribose (Supplementary Figure 9a). Accordingly, the levels of NADP^+^ positively correlated with the levels of NADK in ovarian tumor cells (Supplementary Figure 8c). Finally, we measured the ratio of NADK and NAMPT and observed that the ratio between them closely correlated with NADP^+^/NAD^+^ ratio (Supplementary Figure 8d). Thus these results suggest that biogenesis of NAD^+^ and NADP^+^ plays a key role in determining the NADP^+^/NAD^+^ ratio in cells.

### NADP^+^ is not a donor for ADP-ribosylation

Since NADP^+^ may fit into the catalytic cage of PARP1, we explored if NADP^+^ could participate in the PARP1-dependent PARylation. We generated recombinant NADK protein that can transfer phosphate moiety from ATP to NAD^+^ to synthesize NADP^+^ (Supplementary Figure 9b). Formation of NADP^+^ by our recombinant protein was confirmed by mass spectrometry (Supplementary Figure 9c).

**Fig. 2:**
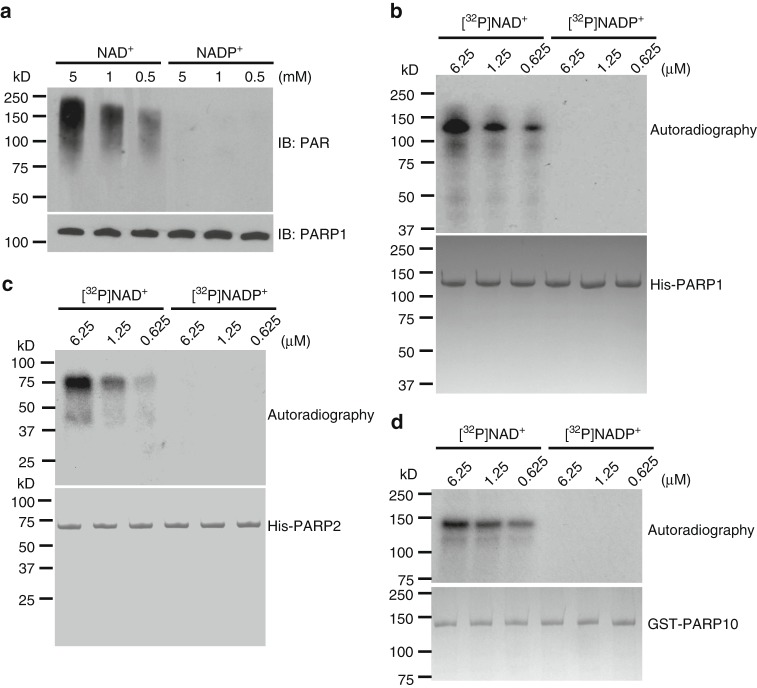
NADP^+^ is not a donor for poly(ADP-ribose) polymerase (PARP)-mediated ADP-ribosylation. **a**, **b** PARP1 cannot use NADP^+^ for PARylation. PARP1-mediated in vitro PARylation assays were performed including recombinant HIS-PARP1, ssDNA, NAD^+^, or NADP^+^. Auto-PARylation of PARP1 was examined by western blotting. 5% of recombinant PARP1 in each sample was extracted before the reaction and was examined as the loading control (**a**). [^32^P]NAD^+^ or [^32^P]NADP^+^ was included in the in vitro PARylation assay. Auto-PARylation of PARP1 was examined by autoradiography (**b**). Coomassie staining of HIS-PARP1 was shown as the loading control. **c** NADP^+^ cannot serve as the donor for PARP2-mediated PARylation. The in vitro PARylation was performed using recombinant HIS-PARP2, ssDNA, and [^32^P]NAD^+^ or [^32^P]NADP^+^. Auto-PARylation of PARP2 was examined by autoradiography. Coomassie staining of HIS-PARP2 was shown as the loading control. **d** NADP^+^ cannot serve as the donor for PARP10-mediated MARylation. Auto-MARylation of PARP10 was examined by autoradiography. Coomassie staining of GST-PARP10 was shown as the loading control

To recapitulate DNA damage-induced PARylation, we performed an in vitro PARylation assay in presence of recombinant PARP1, single-stranded DNA (ssDNA), and NAD^+^ or NADP^+^. The ssDNA in the in vitro assay was used to activate PARP1. As expected, with NAD^+^, PARP1 quickly synthesized PAR chains and autoPARylated itself. In addition, the levels of autoPARylation were increased with increase in the levels of NAD^+^ (Fig. 2a). However, with NADP^+^ alone, PARP1 could not synthesize PAR. Usually, the ADPr moieties in a PAR chain are linked by α(1→2)  O-glycosidic bond between distal ribose and ribose on the adenine side. The phosphate group at 2' position of the ribose next to adenine may suppress the elongation of PAR. Thus we examined whether NADP^+^ could generate MARylation or terminal ADPr residue in the PAR chains. We used ^32^P-NADP^+^ to perform in vitro PARylation assay. Again, autoradiography results show that PARP1 failed to use NADP^+^ as substrate to catalyze ADP-ribosylation (Fig. 2b).

Besides PARP1, other PARPs such as PARP2 and PARP10, also participate in DNA damage-induced ADP-ribosylation with PARP2 catalyzing PARylation and PARP10 catalyzing MARylation^18,19,23^. Thus we wondered whether NADP^+^ could be used by these PARPs for ADP-ribosylation. However, neither PARP2 nor PARP10 could use NADP^+^ for ADP-ribosylation (Fig. 2c, d), suggesting that NADP^+^ cannot contribute ADPr moiety for PARP-dependent ADP-ribosylation.

### NADP^+^ suppresses ADP-ribosylation in vitro

**Fig. 3:**
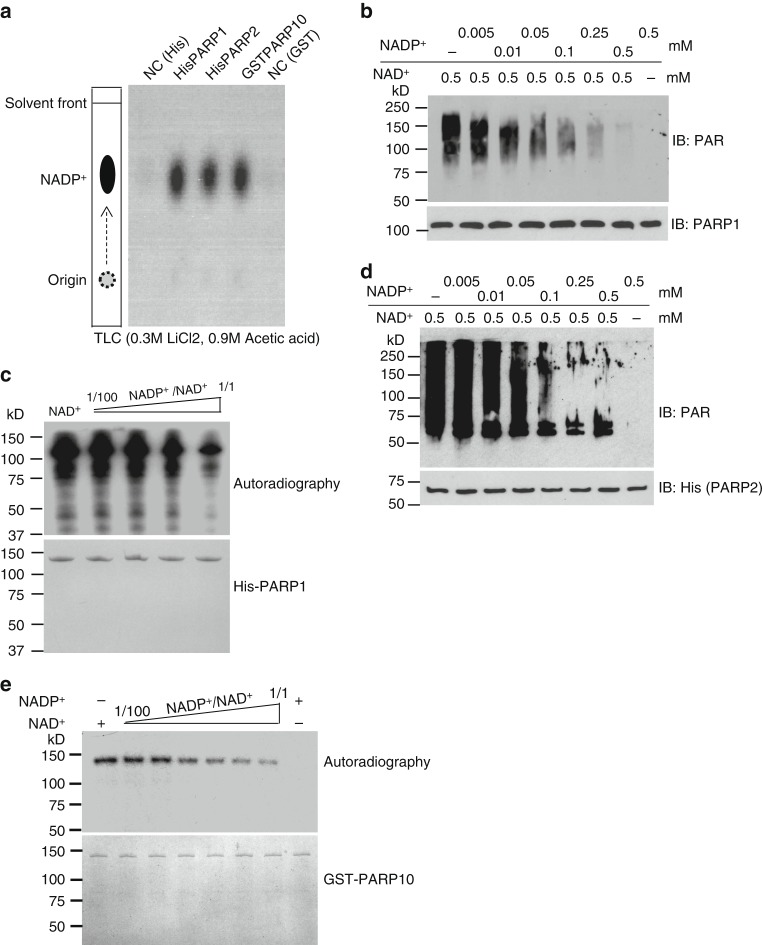
NADP^+^ suppresses poly(ADP-ribose) polymerase (PARP) activity in vitro. **a** PARPs can bind to NADP^+^. HIS-PARP1, HIS-PARP2, and GST-PARP10 were immobilized on Ni Sepharose and glutathione *S*-transferase (GST) beads respectively, followed by incubating with [^32^P]NADP^+^. [^32^P]NADP^+^ was heat-released from Ni Sepharose or GST beads and examined by thin-layer chromatography. As the negative controls (NC), Ni Sepharose or GST beads without recombinant PARP proteins was incubated with [^32^P]NADP^+^. **b**, **c** NADP^+^ suppresses PARP1’s activity in vitro. In vitro PAPR1-mediated PARylation assay was performed using different ratio of NADP^+^/NAD^+^. Auto-PARylation of PARP1 was examined by western blotting (**b**). [^32^P]NAD^+^ was used as the donor in PARP1-mediated in vitro PARylation assay. The auto-PARylation of PARP1 was examined by autoradiography. Coomassie staining of His-PARP1 was shown as the loading control (**c**). **d** NADP^+^ suppresses PARP2 in vitro. Auto-PARylation of PARP2 was examined by western blotting with the indicated antibody. **e** NADP^+^ suppresses PARP10 in vitro. Auto-MARylation level of PARP10 was examined by autoradiography

Although NADP^+^ could not be used for ADP-ribosylation, structure modeling analyses indicate that NADP^+^ fits well into the catalytic cages of PARPs. Based on this observation, we performed in vitro binding assay and found that PARP1, PARP2, and PARP10 indeed were able to bind NADP^+^ (Fig. 3a). Using isothermal titration calorimetry (ITC) assays, we measured the binding affinity between PARP1 and NADP^+^ with the dissociation constant (Kd) value ~39 μM (Supplementary Figure 10). Unfortunately, we could not accurately measure the Kd value between PARP1 and NAD^+^ using ITC. This is due to the fact that PARP1 catalyzes PARylation using NAD^+^ even in the absence of DNA^55^. In addition, it has been reported that the the affinity value (Km) of PARP1 with NAD^+^ ranges from 50 to 250 μM in the in vitro PARylation assays^55,56^. As PARP1-mediated PARylation is a one-step and irreversible reaction in vitro, Km values would be close or even equal to the Kd values. These results indicate that NADP^+^ can outcompete NAD^+^ in PARP1-dependent PARylation at least in vitro.

To test this hypothesis, we performed PARP1-mediated PARylation assays with both NAD^+^ and NADP^+^. Notably, PARP1-mediated PAR synthesis was reduced remarkably with increase in NADP^+^ concentration (Fig. 3b). We further validated these results using ^32^P-NAD^+^ and observed that PARylation inhibition was directly proportional to the levels of unlabeled NADP^+^ (Fig. 3c). Likewise, NADP^+^ suppressed ADP-ribosylation mediated by either PARP2 or PARP10 (Fig. 3d, e). Collectively, these results suggest that, owing to the similar chemical structure, NADP^+^ competes with NAD^+^ for the binding to PARP catalytic site and antagonistically suppresses ADP-ribosylation.

Since our results suggested that NADP^+^ can suppress PARP activity, we wondered whether the two reduced forms of NAD^+^ derivatives including NADH and NADPH can exert similar inhibition effects. We observed that the two reduced forms lacked the ability to suppress the enzymatic activity of PARP1 in the in vitro PARylation assays. These results suggest that the additional hydrogen atom in the NAM ring of NADH and NADPH abolishes the interaction with the catalytic site of PARP1 (Supplementary Figure 11).

### NADP^+^ negatively regulates PARylation

**Fig. 4:**
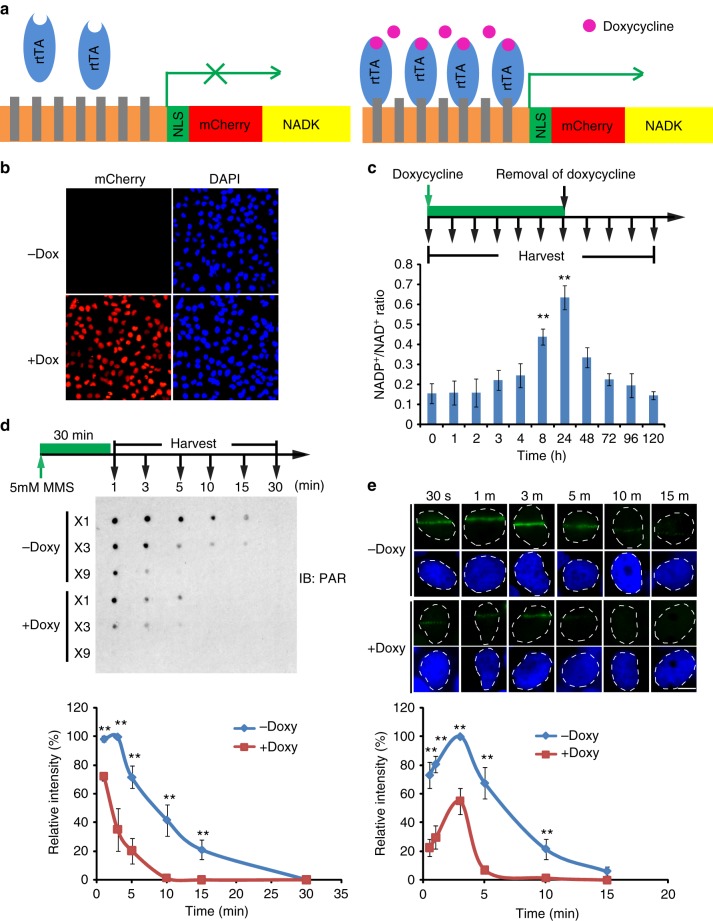
NADP^+^ negatively regulates DNA damage-induced PARylation in cells. **a** The schematic diagram shows the tetracycline-inducible expression of NADK. A nuclear localization signal (NLS) and mCherry were added at the N-terminus of NADK. The Tet-on system is from Addgene. **b** Doxycycline induces the expression of NADK (mCherry) in U2OS cells. **c** The NADP^+^/NAD^+^ ratio in U2OS cells is significantly increased following doxycycline induction. Three independent experiments were performed. Data are presented as mean ± SD. **p < 0.01. Data were analyzed using two-tailed unpaired Student’s *t* tests. **d** High level of NADP^+^ suppresses the DNA damage-induced PARylation. The cells were treated with 10 mM methyl methanesulfonate. Dot blotting was performed to examine PARylation at the indicated time points after DNA damage. The samples were also diluted three times (3×) or 9 times (9×) in the same dot blotting to avoid sample loading variation. Three independent experiments were performed. Data are presented as mean ± SD, **p < 0.01. Data were analyzed using two-tailed unpaired Student’s *t* tests. **e** Kinetics of PARylation at DNA lesions. The cells were treated with laser microirradiation. PARylation at the laser stripes was examined by immunofluorescence staining with anti-PAR antibody. Representative cells at different time points are shown. The relative signal strength of PAR was summarized from three independent experiments and at least 15 cells at each time point in each experiment. Data are presented as mean ± SD. Bars: 10 μM. **p < 0.01. Data were analyzed using two-tailed unpaired Student’s *t* tests

To further investigate the role of NADP^+^ in ADP-ribosylation, we established a system in which doxycycline induction drives the expression of NADK in the nucleus of U2OS cell (Fig. 4a and Supplementary Figure 12). As a control, we mutated the catalytic residue in NADK and generated an enzymatically dead protein (the D148N mutant) (Supplementary Figure 12a). NADK and the D148N mutant were fused with a mCherry tag to confirm the expression. Upon doxycycline treatment, both NADK and NADK mutant expression was achieved in >90% of the cells (Fig. 4b, Supplementary Figure 12b and c). The nuclear expressed NADK quickly phosphorylated NAD^+^ into NADP^+^ and significantly increased the NADP^+^/NAD^+^ratio. In contrast, the D148N failed to convert NAD^+^ to NADP^+^ (Supplementary Figure 12d). Upon the removal of doxycycline, NADP^+^/NAD^+^ ratio gradually reduced back to the control levels (Fig. 4c), suggesting that doxycycline-induced NADK functions as a switch to balance the levels of NADP^+^ and NAD^+^. With this switch, not only NAD^+^, the donor for ADP-ribosylation, is reduced but also NADP^+^, the antagonist for ADP-ribosylation, is increased. Thus conversion of NAD^+^ to NADP^+^ by NADK may double the suppression effect on ADP-ribosylation.

Using this molecular switch, we examined the role of NADP^+^ in DNA damage-induced PARylation. We treated the U2OS cells with MMS to induce DNA damage and examined DNA damage-induced PARylation. In absence of doxycycline induction, MMS treatment led to a quick increase in DNA damage-induced PARylation, which peaked within 1–3 min. The PAR chains were rapidly degraded by dePARylation enzymes within 30 min. In contrast, doxycycline treatment induced the expression of wild-type NADK, which in turn increased NADP^+^/NAD^+^ ratio with a concurrent suppression of PARylation (Fig. 4d, Supplementary Figure 12e). To further validate these results, we treated the cells with laser microirradiation. In agreement, we observed that, in absence of doxycycline, PARylation was detected by anti-PAR antibody at DNA lesions. However, with the doxycycline induction, PARylation was markedly suppressed at the sites of DNA damage (Fig. 4e). Taken together, these results suggest that NADP^+^ upregulation suppresses ADP-ribosylation in cells, which is also consistent with our observation in the panel of ovarian cancer cells (Fig. 1b, e).

### NADP^+^ suppresses early DNA damage response

**Fig. 5:**
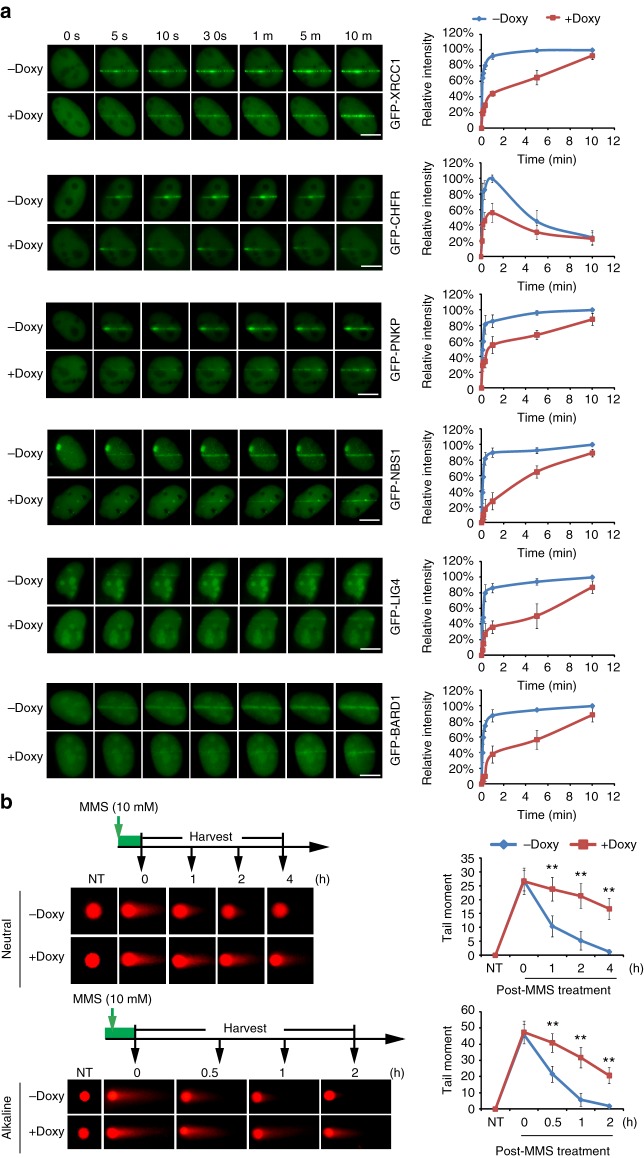
NADP^+^ suppresses the PARylation-dependent early DNA damage response. **a** Doxycycline-induced NADK suppresses the recruitment of the PARylation-dependent DNA damage response. Twenty-four hours after induction, the recruitment kinetics of XRCC1, CHFR, PNKP, NBS1, BARD1, and LIG4 were examined with live cell imaging. The relative intensity on the laser stripes were quantified and summarized at the right panel (mean ± SD, from 15 cells at each time point in each experiment). The highest intensity on the laser stripes was calculated as 100% in each cell, and the kinetics of the recruitment were plotted. Three independent experiments were performed. Bars: 10 μM. **b** High level of NADP^+^ impairs DNA damage repair. The cells were treated with methyl methanesulfonate (10 mM, 30 min). The kinetics of double-strand break and single-strand break repair was examined by neutral and alkaline comet assays, respectively. Representative comet tails at different time points are shown. The tail moments were summarized from at least 50 cells at each time point in each experiment, and three independent experiments were performed. NT non-treated. **p < 0.01. Data were analyzed using two-tailed unpaired Student’s *t* tests

Previous studies have demonstrated that PARylation acts as an early recruitment signal for various DNA damage repair factors at DNA lesions, i.e. single-strand breaks (SSBs) and double-strand breaks (DSBs)^11^. To date, several classes of PAR-binding motifs have been identified in DNA damage repair machineries, such as PAR-binding zinc-finger (PBZ) domain, MACRO domain, WWE domain, BRCA1 C-terminus (BRCT) domain, forkhead-associated (FHA) domain, OB-fold domain, RRM domain, etc^11,29^. Since NADP^+^ negatively regulates PARylation during DNA damage response, we asked whether PARylation-dependent early recruitment of DNA damage repair factors is affected by NADP^+^. We treated cells with laser microirradiation to induce DNA lesions and monitored the early recruitment of SSB repair machineries including XRCC1 and PNKP along with DSB repair machineries, including NBS1, CHFR, LIG4, and BARD1. All these DNA damage repair factors contain PAR-binding motifs and are recruited to the sites of DNA damage within few seconds^29^. Consistent with our previous observation, all these DNA damage repair factors were recruited to the sites of DNA damage quickly and stayed at the DNA lesions for a prolonged time. However, when PARylation was suppressed by doxycycline induction, the early recruitment of these DNA damage repair factors was remarkably suppressed and the kinetics of their recruitment slowed down (Fig. 5a). Moreover, the overall accumulation of these DNA damage response factors was also reduced (Fig. 5a). In contrast, overexpression of the D148N mutant upon doxycycline induction failed to suppress the recruitment of DNA damage repair machinery (Supplementary Figure 13a), suggesting that the suppression in the recruitment of DNA damage repair factors by NADK depends on its enzymatic activity.

Since these DNA damage repair factors play an important role during SSB and DSB repair, suppression of the early and fast recruitment of these repair factors could affect DNA damage repair. Thus we also examined DNA damage repair using single-cell gel electrophoretic comet assay under alkaline as well as neutral conditions. Compared to the control cells, we observed longer comet moments in the cells with wild-type NADK, but not the D148N mutant, overexpression upon doxycycline induction, indicating that conversion of NAD^+^ to NADP^+^ impairs DNA damage repair (Fig. 5b, Supplementary Figure 13b).

### NADP^+^ sensitizes cancer cells to PARP inhibitor

**Fig. 6:**
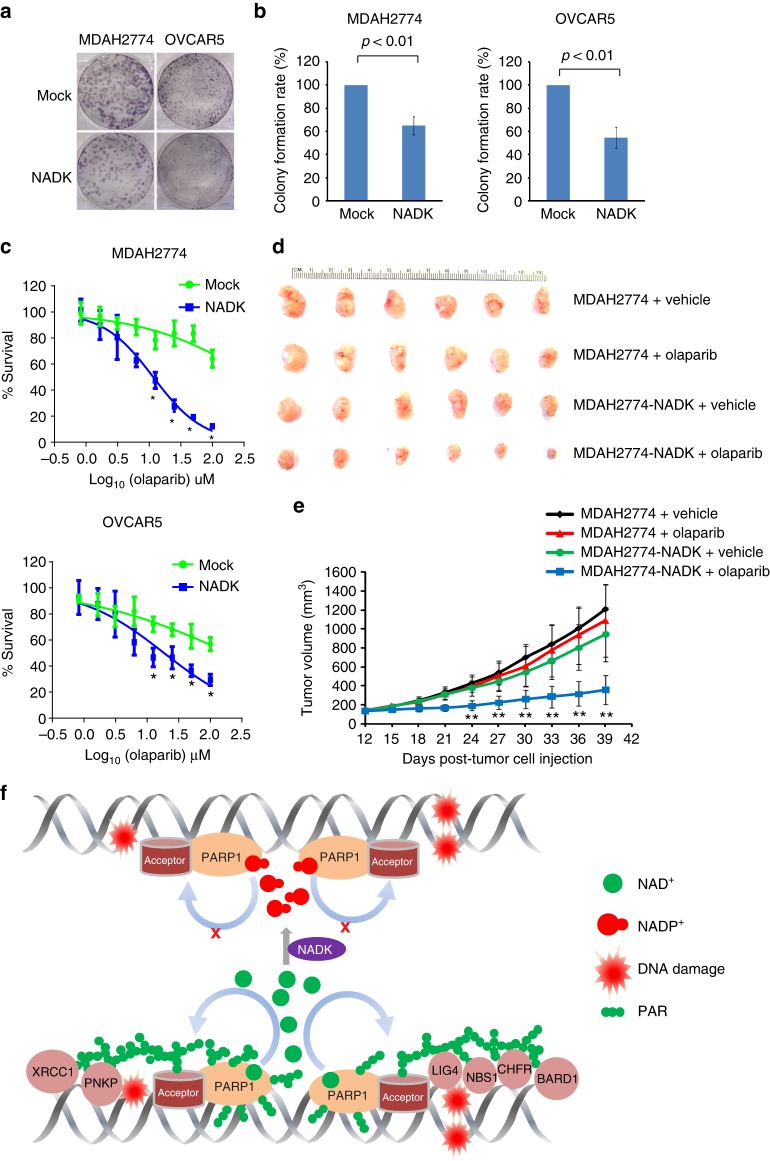
High level of NADP^+^ enhances the sensitivity of cancer cells to chemically synthesized PARP inhibitor. **a**, **b** Increased in the levels of NADP^+^ and olaparib additively suppresses tumor cell growth. MDAH2774 and OVCAR5 with exogenous expression of NADK were examined for colony-formation assays. After treatment with 2 μM olaparib for 2 weeks, the colonies were stained with crystal violet and counted. The results were summarized from three independent experiments. Data are presented as mean ± SD. *p* Value was calculated using two-tailed unpaired Student’s *t* tests. **c** High level of NADP^+^ sensitizes tumor cells to olaparib. MDAH2774 and OVCAR5 with or without the exogenous expression of NADK were treated with olaparib at the indicated concentrations. MTT (3-[4,5-dimethylthiazol-2-yl]-2,5 diphenyl tetrazolium bromide) assays were performed to examine cell viability. The data are summarized from three independent experiments. Data are presented as mean ± SD. *p < 0.05. Data were analyzed using two-tailed unpaired Student’s *t* tests. **d**, **e** High level of NADP^+^ sensitizes xenograft tumors to olaparib. Ovarian cancer xenografts were initiated by subcutaneous inoculation of MDAH2774 cells with or without exogenous expression of NADK in the lower flank of NOD SCID mice as described previously. In all, 5 mg kg^−1^ olaparib was given intraperitoneally daily. Controls were dosed with vehicle only. Both digital photograph (**d**) and tumor growth curve (**e**) shows that exogenous expression of NADK significantly sensitized the MDAH2774 xenograft tumors to olaparib treatment. Data were presented as mean ± SD. **p < 0.01. Data were analyzed using two-tailed unpaired Student’s *t* tests. **f** A model for NADP^+^-mediated suppression of PARylation and DNA damage repair

Here we demonstrate the role of NADP^+^ as an endogenous PARP inhibitor and show the evidence that higher levels of NADP^+^ impair DNA damage repair. Moreover, our screening results in ovarian cancer cells establish a positive correlation between the NADP^+^/NAD^+^ ratio and sensitivity of the cells to PARP inhibitor treatment. To further evaluate the possible implication of NADP^+^ as an endogenous PARP inhibitor in cancer treatment, we chose olaparib-resistant cells, MDAH2774 and OVCAR5, in group IV as a model, which are resistant to olaparib treatment (Figs. 1a and 6b). We first expressed NADK in MDAH2774 and OVCAR5 cells to upregulate the levels of NADP^+^. Ectopic expression of wild-type NADK, but not the D148N mutant, significantly increased the sensitivity of these cells to olaparib treatment in clonogenic assays (Fig. 6a, b, Supplementary Figure 13c). Dose course assays show that upregulated NADP^+^ functions together with olaparib to additively suppress the growth of these cancer cells (Fig. 6c, Supplementary Figure 13d). The inhibitory effects of NADP^+^ were further examined in murine xenograft models. MDAH2774 cells with or without the ectopic expression of NADK were subcutaneously injected into the lower flank of the NOD SCID mice. All mice formed a tumor at the injection site at around 10 days. The NOD SCID mice bearing tumor xenografts were treated with olaparib 5 mg kg^−1^ or the vehicle daily by intraperitoneal injection. Consistent with the in vitro findings, MDAH2774 tumor xenograft was insensitive to olaparib treatment (Fig. 6d, e). In contrast, ectopic expression of NADK markedly sensitized MDAH2774 tumor xenografts to olaparib treatment.

To further validate the role of NADK in cancer treatment, we performed an in vivo assays by using another ovarian cancer cell line HEY, which has been listed in group III. These cells were more sensitive to PARP inhibitor than those in group IV in vitro (Fig. 1a). Consistently, HEY cells were more sensitive to PARP inhibitor than MDAH2774 cells in murine xenograft models. In agreement, NADK overexpression in HEY cells increased their sensitivity to olaparib treatment (Supplementary Figure 14). These results further confirmed our observation with HEY cells in the murine xenograft model. Taken together, our results show that NADP^+^ (endogenous PARP inhibitor) and olaparib (chemically synthesized PARP inhibitor) additively suppress cancer cell growth.

## Discussion

In this study, we demonstrated that NADP^+^ functions as an endogenous PARP inhibitor. We also show that higher ratio of NADP^+^/NAD^+^ in cells impairs ADP-ribosylation and its associated DNA damage response. Moreover, our results reveal that higher sensitivity of ovarian cancer to PARP inhibitors correlates with their higher NADP^+^/NAD^+^ ratio.

NADP^+^ is recognized by PARPs due to its structural resemblance with NAD^+^. However, PARPs could not use NADP^+^ as a substrate for ADP-ribosylation. Thus NADP^+^ competes with NAD^+^ as an antagonist for the binding site in PARPs and negatively regulates ADP-ribosylation. Interestingly, chemically synthesized PARP inhibitors use similar mechanism to block PARylation. PARP inhibitors occupy the catalytic cages of PARP1 and PARP2 and suppress PARylation^37,38^. However, different from chemically synthesized PARP inhibitors, NADP^+^ not only suppresses PARP1 and PARP2 but also including PARP10 and possibly other PARP family enzymes. Thus NADP^+^ likely functions as a universal endogenous PARP inhibitor.

Compared to NAD^+^, NADP^+^ has an additional phospho-group at 2' position of the ribose sugar at adenine side. The phospho-group blocks 1, 2-glycosidic bond formation between the ribose sugars, thus abolishing the linear PAR chain formation. However, besides the linear chains, PAR also contains branched chains that are linked by distal ribose sugars in two ADPr units. It remains elusive why NADP^+^ could not be used for the formation of branched chains by PARPs. In addition, NADP^+^ can neither be used for the terminal residue of PAR nor in the MARylation by PARP family enzymes. Future structural analysis will reveal the molecular details of ADP-ribosylation inhibition by NADP^+^.

NADP^+^ is generated from NAD^+^ by NADK, which is also a potential target for cancer chemotherapy^57,58^. NADK not only reduces the level of NAD^+^, the donor for ADP-ribosylation, but also increases the level of NADP^+^, the inhibitor of ADP-ribosylation. We carefully measured the concentration of NAD^+^ after doxycycline induction in our cellular system (Fig. 4a). We found that ~25% of NAD^+^ was converted by NADK into NADP^+^ 72 h post doxycycline induction (Supplementary Figure 15a). Thus slight shifting in the NAD^+^ and NADP^+^ balance by NADK doubles the inhibitory effects for ADP-ribosylation (Fig. 6f). To further partition the effect caused by the reduction of NAD^+^ and the increase in NADP^+^, we treated the U2OS cells with FK866, a potent NAMPT inhibitor. We found that U2OS cells treated with 2 nM FK866 for 72 h reduced the levels of NAD^+^ by ~25% of cellular NAD^+^. With such reduction of NAD^+^, it is insufficient to affect the DNA damage-induced PARylation, recruitment of DNA repair factors, and DNA damage repair (Supplementary Figure 15). Thus NAD^+^ reduction and NADP^+^ increase cooperatively suppress PARylation and PARylation-dependent DNA damage repair.

Since NADP^+^ shares similar molecular mechanism of PARP inhibition as chemically synthesized PARP inhibitors, NADP^+^, an endogenous PARP inhibitor, additively functions with PARP inhibitors to suppress tumor growth by abolishing DNA damage repair. Thus higher level of NADP^+^ sensitizes tumor cells to PARP inhibitor treatment. Over recent years, PARP inhibition has demonstrated promising potential for targeting cancers with defective DSB repair, including ovarian and breast cancers^39,59^. Olaparib is one of the PARP inhibitors approved by US FDA for treating advanced ovarian cancer associated with BRCA mutations^60^. However, <50% cancer patients with BRCA mutations respond to the PAPR inhibitor treatment^43,44,61^. Moreover, clinical trials also show that PARP inhibitor treatment may be effective even in some cancer patients without BRCA mutations^45,46^. Likewise, we found that several ovarian cancer cell lines with wild-type *BRCA* genes are sensitive to olaparib treatment (Fig. 1a). Thus our study may reveal NADP^+^ as an important biomarker for the evaluation of PARP inhibitor treatment and help in extending the treatment beyond the tumors without BRCA mutations. Moreover, increasing the level of NADP^+^ may also be considered as a therapeutic approach for cancer patients as higher levels of NADP^+^ will sensitize tumor cells to chemically synthesized PARP inhibitor.

Besides being a donor for ADP-ribosylation, NAD^+^ also acts as a crucial cofactor in many biological processes, especially during metabolism as a proton acceptor^62,63^. Moreover, NADP^+^ also performs similar functions as NAD^+^^63^. We observed that increasing the level of NADP^+^ by NADK did not produce any noticeable changes in metabolism, such as in redox reactions involving glucose and glutamine metabolism (Supplementary Figure 16 and 17). In addition, NAD^+^ also functions as an acetyl group acceptor during sirtuin-dependent deacetylation^64–66^. However, upregulation of NADP^+^ in our system did not affect the acetylation status of histone H3K56 and H4K16, i.e., two major targets of sirtuins^67,68^ (Supplementary Figure 18). Thus upregulation of NADP^+^ may be specifically used to target ADP-ribosylation pathway in future cancer therapies.

## Methods

### Antibodies

Rabbit anti-NADK antibody (#15548-1-AP, 1:2000 dilution) was purchased from Proteintech Group, Inc. Mouse anti-β-actin monoclonal antibody (AC-15) (#A1978, 1:10,000 dilution) was purchased from Sigma. Mouse anti-PAR monoclonal antibody (#4335-MC-100, 1:2000 dilution) was purchased from Trevigen. Anti-poly-ADPr-binding reagent (#MABE1031, 1:500 dilution) was purchased from Millipore. Rabbit anti-H3 polyclonal antibody (#06-755, 1:2000 dilution), Rabbit anti-H4 polyclonal antibody (#07-108, 1:2000 dilution), and Rabbit anti-H4K16ac polyclonal antibody (#07-329, 1:2000 dilution) were purchased from Millipore. Rabbit anti-H3K56ac polyclonal antibody (#4243, 1:2000 dilution), Rabbit anti-PARP1 monoclonal antibody (#9532, 1:2000 dilution), and mouse anti-His tag monoclonal antibody (27E8) (#2366, 1:2000 dilution) were purchased from Cell Signaling Technology.

### Ovarian cancer cell lines

The 20 ovarian cancer cell lines used in this study are listed in Fig. 1a. ML3 and ML10 are cystadenoma-derived cell lines, which were generated in Dr. Louis Dubeau laboratory. Cell lines MCV39 and MCV50 are derived from ML10. A2780 and A1847 are epithelial ovarian cancer cell lines that were derived from patients prior to treatment. OVCAR-3, SKOV3, and MDAH2774 have been cultured from malignant ascites from patients with adenocarcinoma. HOC-1, HOC-7, and HEY are serous ovarian carcinoma cell lines. OVCA429 cell line was established from freshly isolated ascites or tumor explants from patients with late-stage ovarian adenocarcinomas with distinct characteristics. PEO1, derived from a BRCA2 mutation [5193C>G (Y1655X)] carrier with ovarian carcinoma, is BRCA2 deficient and sensitive to cisplatin. PEO4, derived from the ascites at the time of relapse with cisplatin resistance, has the secondary mutation [5193C>T (Y1655Y)] and was BRCA2 proficient^50^. Epithelial ovarian cancer cell line, TOV112-D, was derived from an endometrioid carcinoma, which was never exposed to chemotherapy or radiation therapy. OVCAR-4, OVCAR-5, and OVCAR-10 were derived from ovarian adenocarcinoma. OVCAR-4 and OVCAR-10 cells were derived from the patient refractory to cisplatin, and OVCAR-5 was from the patient with advanced ovarian tumor prior to treatment. DOV-13 is an ovarian adenocarcinoma cell line established by E. L. Brown laboratory. All the ovarian cells were cultured in Dulbecco’s modified Eagle’s medium media containing 10% fetal bovine serum, 2 mM L-glutamine, and antibiotics.

### MTT (3-[4,5-dimethylthiazol-2-yl]-2,5 diphenyl tetrazolium bromide) assay

The sensitivity of ovarian cancer cells to olaparib and niraparib was assessed by MTT assay. Ovarian cancer cells were plated in flat bottom 96-well plates at 2000 cells per well (final volume 200 μl per well). Cells were treated with olaparib or niraparib at the indicated concentrations for 7 days. Ten μl of 5 mg ml^−1^ MTT solution in phosphate-buffered saline (PBS) was added into each well for 4 h. After removing the media, 100 μl dimethyl sulfoxide (DMSO) was added to each well to dissolve the formazan crystals. The absorbance at 570 nm was determined using a Biokinetics plate reader (Bio-Tek Instruments, Inc, Winooski, VT, USA). Triplicate wells were assayed for each condition and S.D. was determined.

### Measuring NAD^+^ and NADP^+^ concentration

The concentrations of NAD^+^ and NADP^+^ were measured according to the manufacturer’s recommendations (ECNP-100 and E2ND-100, respectively, BioAssay system). Briefly, one million cells were lysed with 100 μl NADP^+^ extraction buffer. Cell lysates were heated to 60 °C for 5 min After that, 20 μl of NAD^+^ or NADP^+^ assay buffer was added for NAD^+^ or NADP^+^ measurements, respectively. The levels of NAD^+^ and NADP^+^ were measured with lactate dehydrogenase and glucose 6 phosphate dehydrogenase enzymatic cycling methods. The absorbance at 565 nm was determined using a Biokinetics plate reader (Bio-Tek Instruments, Inc, Winooski, VT, USA). Standard NAD^+^ and NADP^+^ were used to prepare standard curve. NAD^+^ and NADP^+^ concentration were calculated according to the standard curve.

### Recombinant PARP protein expression and purification

HIS-tagged human PARP1, PARP2, GST-tagged human PARP10, and GST-tagged human PARP1 catalytic domain (GST-PARP1 CAT, aa. 662–1014) proteins were expressed in *Escherichia coli* BL21. Cells were grown in LB media and induced with 200 μM isopropyl 1-thio-β-d-galactopyranoside at 16 °C for 20 h. Proteins fused to GST were purified using glutathione-Sepharose beads according to the manufacturer’s protocols (GE Healthcare). HIS-tagged proteins were purified by Ni Sepharose 6 Fast Flow according to the manufacturer’s instruction (GE Healthcare). All recombinant proteins were further purified by passing through Superose 6 10/300 GL column (GE Healthcare) in 50 mM sodium phosphate buffer, pH 7.0, and 150 mM NaCl. Expression and purification of all recombinant proteins was analyzed by sodium dodecyl sulfate–polyacrylamide gel electrophoresis (SDS–PAGE) followed by Coomassie staining.

### In vitro PARP activation assays

The in vitro PARP activation assays were performed according to the previous work with some modifications^69^. Briefly, the reaction mixture (15 μl) containing 150 nM recombinant human PARP protein, 100 mM Tris-HCl pH 7.8, 10 mM MgCl_2_, 10 mM dithiothreitol, 50 mg ml^−1^ octameric oligonucleotide GGAATTCC (for PARP1- and PARP2-mediated PARylation), and NAD^+^ or [^32^P]NAD^+^ at the indicated concentrations were assembled on ice. The reaction mixtures were incubated at 37 °C for 60 min. Reactions were terminated by the addition of 10 μl SDS-PAGE sample buffer followed by heating at 95 °C for 5 min.

### Isothermal titration calorimetry

ITC was carried out at room temperature with a Nano ITC (TA Instrument). Proteins were dialyzed extensively into the buffer containing 10 mM Na2HPO4 (pH 7.5) and 100 mM NaCl at the final concentrations of 20 µM. The ligand, NADP^+^, in the injection syringe were also diluted by the same buffer at final concentration of 4 mM. Recombinant catalytic domain of PARP1 was titrated with 17 injections (2 µl per injection) of NADP^+^ into the sample cell. The dissociation constant (Kd) was calculated by using the analytic software from the manufacturer.

### Comet assay

Single-cell gel electrophoretic comet assays were performed under alkaline or neutral conditions to test DNA SSBs or DSBs. DNA break repair was analyzed by single-cell agarose gel electrophoresis. U2OS cells expressing NADK were incubated with 10 mM MMS at 37 °C for 30 min. For cell lysis, the slides were immersed in neutral lysis solution (2% sarkosyl, 0.5 M EDTA, 0.5 mg ml^−^^1^ proteinase K, pH 8.0) or in alkaline solution (1.2 M NaCl, 100 mM Na_2_ EDTA, 0.1% sodium lauryl sarcosinate, 0.26 M NaOH, pH > 13) overnight at 37 °C. On the second day, after electrophoresis at 15–20 V for 25 min (0.6 V cm^−1^), the slides were stained for 20 min with 2.5 g ml^−1^ propidium iodide and viewed in a fluorescence microscope. The comet tail moment was analyzed by the CometScore software.

### Laser microirradiation and immunostaining

For laser microirradiation, cells were grown on 35-mm glass bottom dishes (MatTek Corporation). Laser microirradiation was performed on OLYMPUS-IX71 inverted fluorescence microscope with a Micropoint® Laser Illumination and Ablation System (Photonic Instruments). The GPF strips were recorded at the indicated time points and then analyzed with the ImageJ software. For the time course analysis of laser microirradiation, samples were subjected to continuous microirradiation along certain paths within the indicated time interval. For immunostaining, cells were fixed with 3% paraformaldehyde for 10 min and permeabilized with 0.5% Triton X-100 in PBS for 5 min at room temperature. Samples were blocked with 5% goat serum and then incubated with the primary antibody for 1 h. Samples were washed for three times and incubated with the secondary antibodies for 30 min The coverslips were mounted onto glass slides and visualized with OLYMPUS-IX71 inverted fluorescence microscope. All the images were acquired with the cellSens standard (Version 1.3) software under OLYMPUS IX71 inverted fluorescence microscope equipped with an UPlanSApo ×60/1.35 oil immersion objective at room temperature. Identical contrast and brightness adjustment were used on images for all experiments.

### Purification of cellular PAR and dot blotting

Cells were lysed with 10 mM Tris-HCl (pH 8.5), 2 mM MgCl_2_, and 10% SDS solution following 10 mM MMS (Sigma) treatment for 30 min. When lysed with PARG inhibitor, 1 µM ADP-HPD and 5 µM GLTN were included. Next, the samples were incubated 2 h with additional 0.1% proteinase K (Thermo Scientific). The samples were then extracted with equal volumes of phenol/chloroform and chloroform, and the aqueous layer was obtained. PAR was recovered from the aqueous layer with ethanol precipitation by adding 0.1 volumes of 3 M NH_4_Ac (pH 9.0) and 2 volumes of ethanol at room temperature. After centrifugation at 16,000 × *g* for 30 min, the pellet was washed with 70% ethanol and dried, and the pellet was resuspended into 20 µl deionized water for dot blotting.

Purified PAR was dotted on Hybond-N+ nitrocellulose membrane (Amersham Pharmacia Biotech). After drying at 55 °C, the membrane was blocked with 10% non-fat milk for 1 h at room temperature followed by 2-h incubation with anti-PAR antibodies at room temperature. After three consecutive 10-min washes with TBST, the membrane was incubated with horseradish peroxidase-conjugated goat-anti-rabbit secondary antibody for 1 h. The membrane was washed again for three times with TBST and developed using the Enhanced Chemi-Luminescence plus (ECL+) detection system (GE Healthcare).

### Enzyme-linked immunosorbent assay (ELISA)

NAMPT and NADK protein expression levels were quantified using ELISA according to the manufacturer's protocols. The NAMPT intracellular ELISA kit was purchased from ENZO Life Sciences, Inc (#AG-45A-0006EK-KI01). NADK ELISA kit was purchased from Antibodies-online (#ABIN423195). The relative protein expression levels were calculated by normalization to total protein.

### HR assay

The HR assay has been well established^70^. HR reporter DR-GFP plasmid was kindly provided by Dr. Jeremy Stark. Twenty ovarian cell lines were transfected with DR-GFP plasmids and selected with puromycin for 3 days. DR-GFP-expressing cells were infected with adenovirus-encoded I-SceI (adeno-I-SceI). Cells were harvested 3 days after infection and subjected to flow cytometric analysis. The GFP-positive cell population was measured. Adenovirus infection efficiency was examined in ovarian cancer cells prior to the HR assays. At a multiplicity of infection of 1000, the infection efficiency was close to 100% with control adeno-GFP. Each experiment has been performed at least three times.

### PARP inhibitor treatment

Olaparib and niraparib were purchased from AdooQ Bioscience. Olaparib was used by diluting 50 mg ml^−1^ stocks in DMSO with 10% 2-hydroxyl-propyl-β-cyclodextrine/PBS such that the final volume administered by intraperitoneal (i.p.) injection was 10 µl g^−1^ of body weight.

### Animal studies

Four–6-week-old female NOD SCID mice (Jackson Laboratory) were used for xenografting studies. Ovarian cancer cells MDAH2774 and HEY with or without ectopic expression of NADK were trypsinized and washed twice in serum-free medium before inoculation in mice. In all, 4 × 10^6^ MDAH2774 or HEY cells resuspended in 100 µl serum-free medium with 100 µl Matrigel (BD Bioscience) were injected subcutaneously into the lower flank of the mice. When the tumors reached a size of ≈150 mm^3^, 5 mg kg^−1^ olaparib was given i.p. daily. Controls were dosed with vehicle only. Tumor growth was monitored every 3 days by taking measurements of the tumor length (*L*) and width (*W*) with a digital caliper until the end of the study when the largest tumor size reached 1500 mm^3^. Tumor volume was calculated as π*LW*^2^/6. Only tumors with diameter of >0.3 cm were considered. All the animal experiments were performed in accordance with National Institute of Health animal use guidelines and protocols after approval by City of Hope Beckman Research Institute Animal Care and Use Committee.

### Statistical analysis

Data were analyzed by the Student’s *t* test. Spearman’s correlation was performed to determine the correlation among IC50 of olaparib, PARylation levels, PARP1 levels, NAMPT protein levels, NADK protein levels, relative NADK/NAMPT ratio, NAD^+^ concentrations, NADP^+^ concentrations, and the NADP^+^/NAD^+^ ratios by using the online software, Wessa, P. (2017), (Free Statistics Software, Office for Research Development and Education, version 1.1.23-r7, http://www.wessa.net/). A difference with a *p* < 0.05 was considered statistically significant.

### Reporting summary

Further information on experimental design is available in the Nature Research Reporting Summary linked to this article.

## Supplementary information

Supplementary Information

Reporting Summary

## Data Availability

Uncropped blots and gels of major figures are shown in Supplementary Figure 19 and all data are available from the corresponding author upon reasonable request.

## References

[1] Gibson BA, Kraus WL (2012). New insights into the molecular and cellular functions of poly(ADP-ribose) and PARPs. Nat. Rev. Mol. Cell Biol..

[2] Ame JC, Spenlehauer C, de Murcia G (2004). The PARP superfamily. Bioessays.

[3] Hottiger MO, Hassa PO, Luscher B, Schuler H, Koch-Nolte F (2010). Toward a unified nomenclature for mammalian ADP-ribosyltransferases. Trends Biochem. Sci..

[4] Vivelo CA, Wat R, Agrawal C, Tee HY, Leung AK (2017). ADPriboDB: the database of ADP-ribosylated proteins. Nucleic Acids Res..

[5] Martello R (2016). Proteome-wide identification of the endogenous ADP-ribosylome of mammalian cells and tissue. Nat. Commun..

[6] Jungmichel S (2013). Proteome-wide identification of poly(ADP-Ribosyl)ation targets in different genotoxic stress responses. Mol. Cell.

[7] Vyas S, Chang P (2014). New PARP targets for cancer therapy. Nat. Rev. Cancer.

[8] Bonfiglio JJ (2017). Serine ADP-ribosylation depends on HPF1. Mol. Cell.

[9] Zhang Y, Wang J, Ding M, Yu Y (2013). Site-specific characterization of the Asp- and Glu-ADP-ribosylated proteome. Nat. Methods.

[10] Altmeyer M, Hottiger MO (2009). Poly(ADP-ribose) polymerase 1 at the crossroad of metabolic stress and inflammation in aging. Aging.

[11] Wei H, Yu X (2016). Functions of PARylation in DNA damage repair pathways. Genomics Proteomics Bioinformatics.

[12] Leslie Pedrioli, D. M. et al. Comprehensive ADP-ribosylome analysis identifies tyrosine as an ADP-ribose acceptor site. *EMBO Rep*. **19**, e45310 (2018). 10.15252/embr.201745310 PMC6073207 29954836

[13] Luo, X. & Kraus, W. L. On par with PARP: cellular stress signaling through poly(ADP-ribose) and PARP-1. *Genes Dev*. **26**, 417–432 (2012). 10.1101/gad.183509.111 PMC3305980 22391446

[14] De Vos M, Schreiber V, Dantzer F (2012). The diverse roles and clinical relevance of PARPs in DNA damage repair: current state of the art. Biochem. Pharmacol..

[15] Dregalla RC (2010). Regulatory roles of tankyrase 1 at telomeres and in DNA repair: suppression of T-SCE and stabilization of DNA-PKcs. Aging.

[16] Kim MY, Zhang T, Kraus WL (2005). Poly(ADP-ribosyl)ation by PARP-1: 'PAR-laying' NAD+ into a nuclear signal. Genes Dev..

[17] Shieh WM (1998). Poly(ADP-ribose) polymerase null mouse cells synthesize ADP-ribose polymers. J. Biol. Chem..

[18] Riccio AA, Cingolani G, Pascal JM (2016). PARP-2 domain requirements for DNA damage-dependent activation and localization to sites of DNA damage. Nucleic Acids Res..

[19] Ame JC (1999). PARP-2, a novel mammalian DNA damage-dependent poly(ADP-ribose) polymerase. J. Biol. Chem..

[20] Menissier de Murcia J (2003). Functional interaction between PARP-1 and PARP-2 in chromosome stability and embryonic development in mouse. EMBO J..

[21] Boehler C (2011). Poly(ADP-ribose) polymerase 3 (PARP3), a newcomer in cellular response to DNA damage and mitotic progression. Proc. Natl Acad. Sci. USA.

[22] Grundy GJ (2016). PARP3 is a sensor of nicked nucleosomes and monoribosylates histone H2B(Glu2). Nat. Commun..

[23] Nicolae CM (2014). The ADP-ribosyltransferase PARP10/ARTD10 interacts with proliferating cell nuclear antigen (PCNA) and is required for DNA damage tolerance. J. Biol. Chem..

[24] Kleine H (2008). Substrate-assisted catalysis by PARP10 limits its activity to mono-ADP-ribosylation. Mol. Cell.

[25] Vyas S (2014). Family-wide analysis of poly(ADP-ribose) polymerase activity. Nat. Commun..

[26] Fouquerel E, Sobol RW (2014). ARTD1 (PARP1) activation and NAD(+) in DNA repair and cell death. DNA Repair (Amst.).

[27] Daniels CM, Ong SE, Leung AK (2015). The promise of proteomics for the study of ADP-ribosylation. Mol. Cell.

[28] Poirier GG, de Murcia G, Jongstra-Bilen J, Niedergang C, Mandel P (1982). Poly(ADP-ribosyl)ation of polynucleosomes causes relaxation of chromatin structure. Proc. Natl Acad. Sci. USA.

[29] Liu C, Vyas A, Kassab MA, Singh AK, Yu X (2017). The role of poly ADP-ribosylation in the first wave of DNA damage response. Nucleic Acids Res..

[30] Osterman, A. Biogenesis and homeostasis of nicotinamide adenine dinucleotide cofactor. *EcoSal Plus* **3**, 10.1128/ecosalplus.3.6.3.10 (2009). 10.1128/ecosalplus.3.6.3.10 PMC4229845 26443758

[31] Garten A, Petzold S, Korner A, Imai S, Kiess W (2009). Nampt: linking NAD biology, metabolism and cancer. Trends Endocrinol. Metab..

[32] Chiarugi A, Dolle C, Felici R, Ziegler M (2012). The NAD metabolome--a key determinant of cancer cell biology. Nat. Rev. Cancer.

[33] Magni G, Orsomando G, Raffaelli N (2006). Structural and functional properties of NAD kinase, a key enzyme in NADP biosynthesis. Mini Rev. Med. Chem..

[34] Ryu, K. W., et al. Metabolic regulation of transcription through compartmentalized NAD(+) biosynthesis. *Science* **360**, eaan5780 (2018). 10.1126/science.aan5780 PMC6465534 29748257

[35] George, A., Kaye, S. & Banerjee, S. Delivering widespread BRCA testing and PARP inhibition to patients with ovarian cancer. *Nat. Rev. Clin. Oncol.* **14**, 284–296 (2016). 10.1038/nrclinonc.2016.191 27958297

[36] Scott CL, Swisher EM, Kaufmann SH (2015). Poly (ADP-ribose) polymerase inhibitors: recent advances and future development. J. Clin. Oncol..

[37] Rouleau M, Patel A, Hendzel MJ, Kaufmann SH, Poirier GG (2010). PARP inhibition: PARP1 and beyond. Nat. Rev. Cancer.

[38] Murai J (2012). Trapping of PARP1 and PARP2 by clinical PARP inhibitors. Cancer Res..

[39] McCabe N (2006). Deficiency in the repair of DNA damage by homologous recombination and sensitivity to poly(ADP-ribose) polymerase inhibition. Cancer Res..

[40] Roy R, Chun J, Powell SN (2011). BRCA1 and BRCA2: different roles in a common pathway of genome protection. Nat. Rev. Cancer.

[41] Bryant HE (2005). Specific killing of BRCA2-deficient tumours with inhibitors of poly(ADP-ribose) polymerase. Nature.

[42] Farmer H (2005). Targeting the DNA repair defect in BRCA mutant cells as a therapeutic strategy. Nature.

[43] Fong PC (2010). Poly(ADP)-ribose polymerase inhibition: frequent durable responses in BRCA carrier ovarian cancer correlating with platinum-free interval. J. Clin. Oncol..

[44] Kaufman B (2015). Olaparib monotherapy in patients with advanced cancer and a germline BRCA1/2 mutation. J. Clin. Oncol..

[45] Ledermann J (2014). Olaparib maintenance therapy in patients with platinum-sensitive relapsed serous ovarian cancer: a preplanned retrospective analysis of outcomes by BRCA status in a randomised phase 2 trial. Lancet Oncol..

[46] Comen EA, Robson M (2010). Poly(ADP-ribose) polymerase inhibitors in triple-negative breast cancer. Cancer J..

[47] Banerjee S, Kaye SB, Ashworth A (2010). Making the best of PARP inhibitors in ovarian cancer. Nat. Rev. Clin. Oncol..

[48] Sandhu SK (2013). The poly(ADP-ribose) polymerase inhibitor niraparib (MK4827) in BRCA mutation carriers and patients with sporadic cancer: a phase 1 dose-escalation trial. Lancet Oncol..

[49] Sisay M, Edessa D (2017). PARP inhibitors as potential therapeutic agents for various cancers: focus on niraparib and its first global approval for maintenance therapy of gynecologic cancers. Gynecol. Oncol. Res. Pract..

[50] Sakai W (2009). Functional restoration of BRCA2 protein by secondary BRCA2 mutations in BRCA2-mutated ovarian carcinoma. Cancer Res..

[51] Stordal B (2013). BRCA1/2 mutation analysis in 41 ovarian cell lines reveals only one functionally deleterious BRCA1 mutation. Mol. Oncol..

[52] Samouelian V (2004). Chemosensitivity and radiosensitivity profiles of four new human epithelial ovarian cancer cell lines exhibiting genetic alterations in BRCA2, TGFbeta-RII, KRAS2, TP53 and/or CDNK2A. Cancer Chemother. Pharmacol..

[53] Sulkowski P. L., et al. 2-Hydroxyglutarate produced by neomorphic IDH mutations suppresses homologous recombination and induces PARP inhibitor sensitivity. *Sci. Transl. Med.* **9**, eaal2463 (2017). 10.1126/scitranslmed.aal2463 PMC5435119 28148839

[54] Steffen JD, Brody JR, Armen RS, Pascal JM (2013). Structural implications for selective targeting of PARPs. Front. Oncol..

[55] Langelier MF, Ruhl DD, Planck JL, Kraus WL, Pascal JM (2010). The Zn3 domain of human poly(ADP-ribose) polymerase-1 (PARP-1) functions in both DNA-dependent poly(ADP-ribose) synthesis activity and chromatin compaction. J. Biol. Chem..

[56] Altmeyer M, Messner S, Hassa PO, Fey M, Hottiger MO (2009). Molecular mechanism of poly(ADP-ribosyl)ation by PARP1 and identification of lysine residues as ADP-ribose acceptor sites. Nucleic Acids Res..

[57] Tedeschi PM (2016). NAD+ kinase as a therapeutic target in cancer. Clin. Cancer Res..

[58] Tsang YH (2016). Functional annotation of rare gene aberration drivers of pancreatic cancer. Nat. Commun..

[59] Patel AG, Sarkaria JN, Kaufmann SH (2011). Nonhomologous end joining drives poly(ADP-ribose) polymerase (PARP) inhibitor lethality in homologous recombination-deficient cells. Proc. Natl Acad. Sci. USA.

[60] Kim G (2015). FDA approval summary: olaparib monotherapy in patients with deleterious germline BRCA-mutated advanced ovarian cancer treated with three or more lines of chemotherapy. Clin. Cancer Res..

[61] Tutt A (2010). Oral poly(ADP-ribose) polymerase inhibitor olaparib in patients with BRCA1 or BRCA2 mutations and advanced breast cancer: a proof-of-concept trial. Lancet.

[62] Verdin E (2015). NAD(+) in aging, metabolism, and neurodegeneration. Science.

[63] Ying W (2008). NAD+/NADH and NADP+/NADPH in cellular functions and cell death: regulation and biological consequences. Antioxid. Redox Signal..

[64] Imai S, Armstrong CM, Kaeberlein M, Guarente L (2000). Transcriptional silencing and longevity protein Sir2 is an NAD-dependent histone deacetylase. Nature.

[65] Michan S, Sinclair D (2007). Sirtuins in mammals: insights into their biological function. Biochem. J..

[66] Landry J (2000). The silencing protein SIR2 and its homologs are NAD-dependent protein deacetylases. Proc. Natl Acad. Sci. USA.

[67] Toiber D (2013). SIRT6 recruits SNF2H to DNA break sites, preventing genomic instability through chromatin remodeling. Mol. Cell.

[68] Vaquero A, Sternglanz R, Reinberg D (2007). NAD+-dependent deacetylation of H4 lysine 16 by class III HDACs. Oncogene.

[69] Masaoka A (2012). HMGN1 protein regulates poly(ADP-ribose) polymerase-1 (PARP-1) self-PARylation in mouse fibroblasts. J. Biol. Chem..

[70] Weinstock DM, Nakanishi K, Helgadottir HR, Jasin M (2006). Assaying double-strand break repair pathway choice in mammalian cells using a targeted endonuclease or the RAG recombinase. Methods Enzymol..

