# Supplementary material for: NADP+ is an endogenous PARP inhibitor in DNA damage response and tumor suppression
Source: Nat Commun. 2019 Feb 11;10:693. doi: 10.1038/s41467-019-08530-5 (PMC6370829; doi:10.1038/s41467-019-08530-5)
Supplement: Supplementary file 1 — Supplementary Information [file 41467_2019_8530_MOESM1_ESM.pdf]

Supplementary Information

NADP$^+$ is an endogenous PARP inhibitor in DNA damage response and tumor suppression

Bian et al.

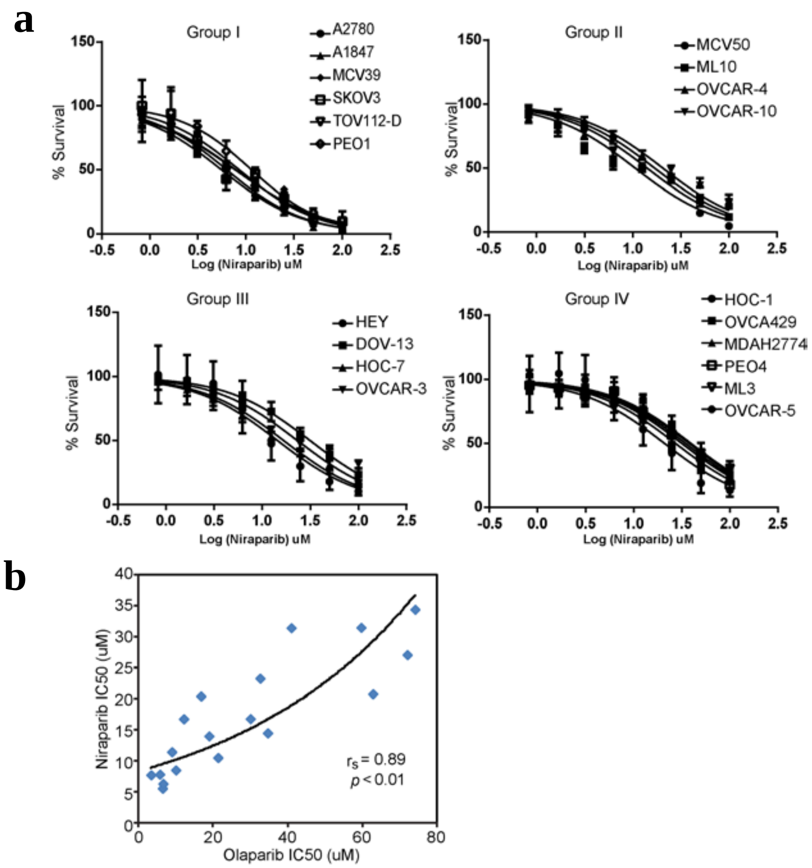

Supplementary Figure 1. The sensitivity of 20 ovarian cancer cell lines to niraparib treatment. (**a)** 20 ovarian cancer cell lines were treated with niraparib at the indicated doses. The sensitivity of the ovarian cancer cells from each group to niraparib was assessed by MTT assays. The data were summarized from three independent experiments. Data were presented as mean±SD. (**b)** Spearman's correlation analysis between IC50 of niraparib and the IC50 of olaparib in 20 ovarian cancer cell lines. Both IC50 of olaparib and IC50 of niraparib were calculated from the MTT assays. *p* value of Spearman's correlation was calculated by R function, cor.test().

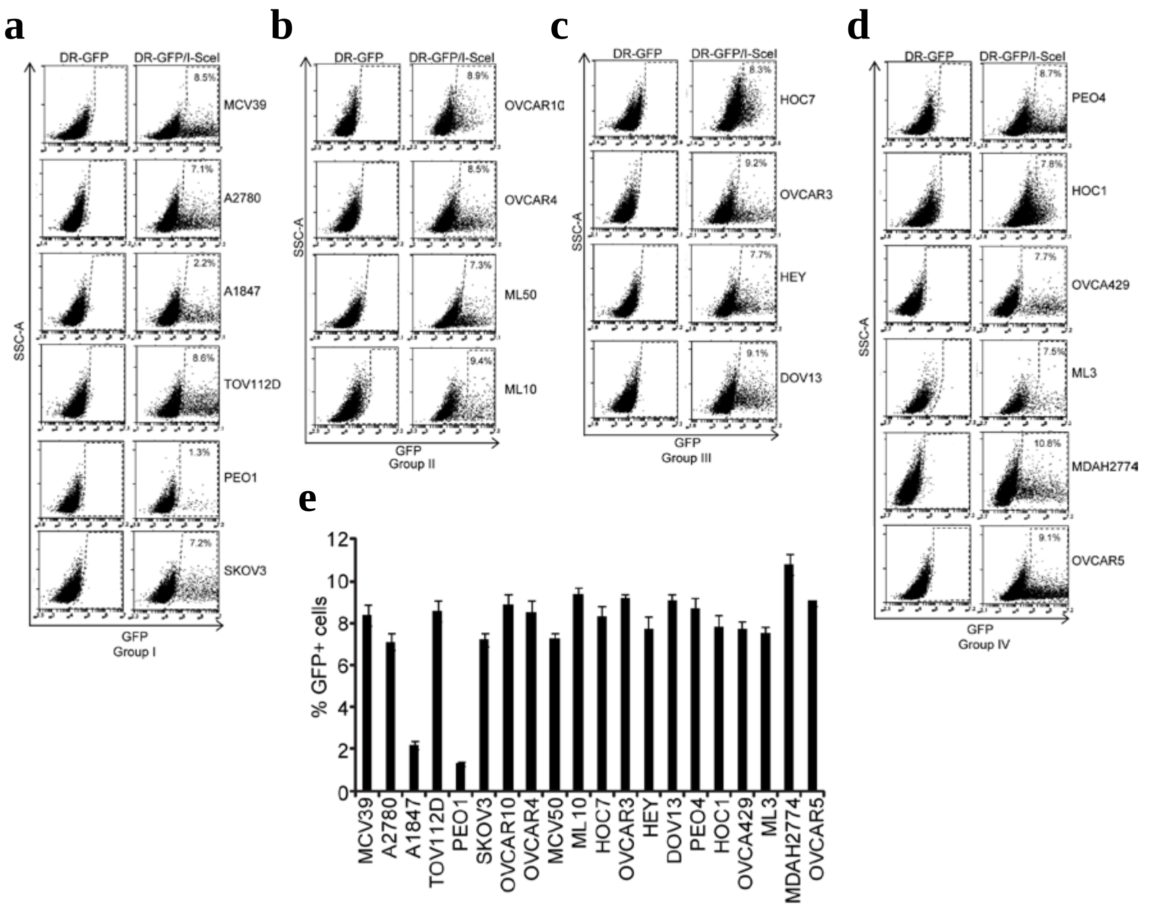

Supplementary Figure 2. HR repair efficiency in 20 ovarian cancer cell lines. (**a**) Group I ovarian cancer cell lines were first transfected with HR reporter DR-GFP and selected in puromycin for 3 days. DR-GFP expressing cells were infected with adenovirus-encoded I-SceI (adeno-I-SceI). Cells were harvested 3 days post infection and subjected to flow cytometry. FSC-A/SSC-A gates of the starting cell population were used to dicriminate between viable cells and cells deribris. Singlet and doublet cells were discriminated using FSC-A/FSC-W gating. HR repair was impaired in A1847 and PEO1 cells due to hypermethylation in BRCA1 promoter and BRCA2 mutation respectively. Other Group I cell lines do not have any obvious HR defect. (**b-d**) In group II, III and IV cell lines, no significant HR defects were observed. (**e**) Percentage of GFP^{+} cells is

summarized from the flow cytometric analyses of 20 ovarian cancer cell lines. Data were presented as mean ± SD of three independent experiments.

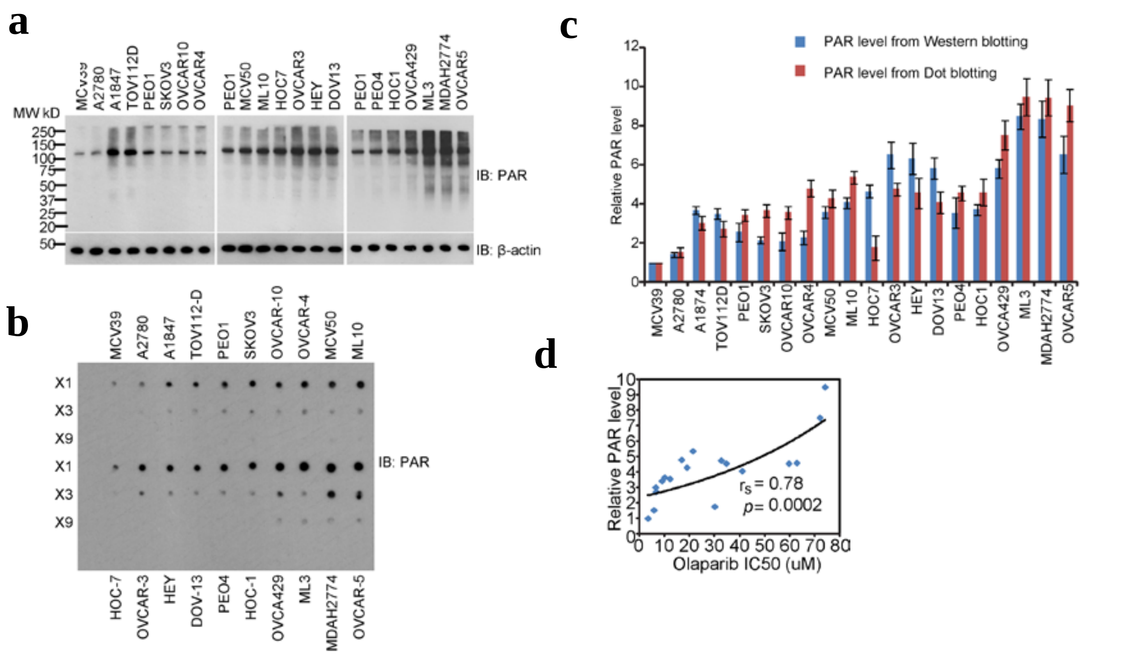

Supplementary Figure 3. DNA damage-induced PARylation in olaparib sensitive cells are markedly lower than that in olaparib insensitive cells. (**a-b**) 20 ovarian cancer cell lines were treated with 10 mM MMS for 30 min. DNA damage-induced PARylation was examined by Western blotting and Dot blotting with PAR-binding reagent (MABE 1031, Millipore) respectively. The samples were diluted three times (x3) or 9 times (x9) in the same Dot blotting to avoid sample loading variation. (**c**) DNA damage-induced PARylation levels were summarized from Western blotting and Dot blotting using ImageJ software. Three independent experiments were performed. Data were presented as mean ± SD. **(d)** Spearman's correlation analysis between DNA damage-induced PARylation levels and the IC50 of olaparib. DNA damage-induced endogenous PARylation levels were summarized from the Dot blotting. IC50 of olaparib was calculated from the MTT assays. DNA damage induced endogenous PARylation levels correlated with IC50 of olaparib. *p* value of Spearman's correlation was calculated by R function, cor.test().

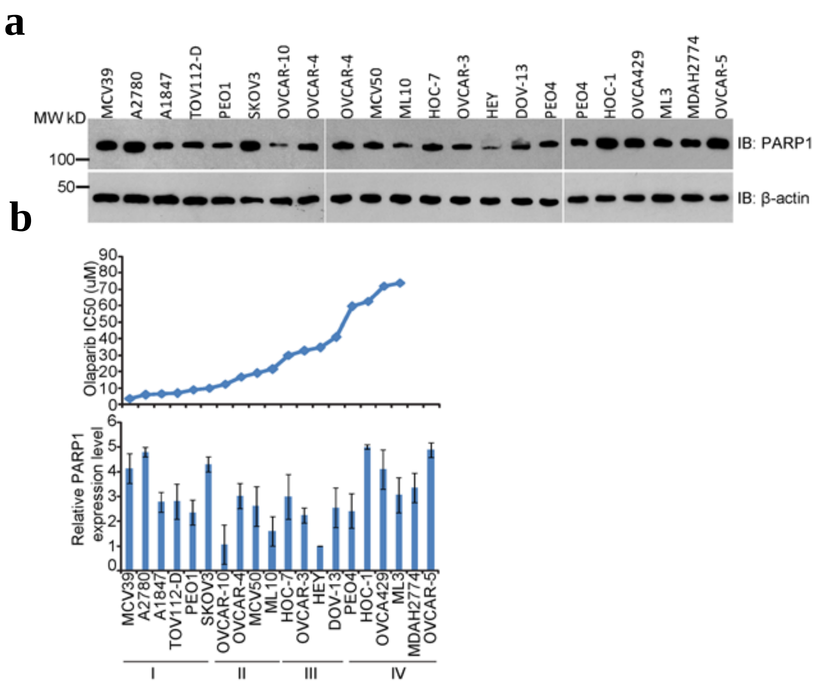

Supplementary Figure 4. Sentivity of ovarian cancer cells to olaparib is not associated with the PARP1 expression levels in ovarian cancer cell lines. (**a**) The expression level of PARP1 was examined in 20 ovarian cancer cell lines by Western blotting. β-actin was shown as the loading control. (**b**) PARP1 expression levels were summarized from the Western blotting results by using ImageJ software. IC50 value of olaparib was calculated from the MTT assays. Three independent experiments were performed. Data were presented as mean ± SD.

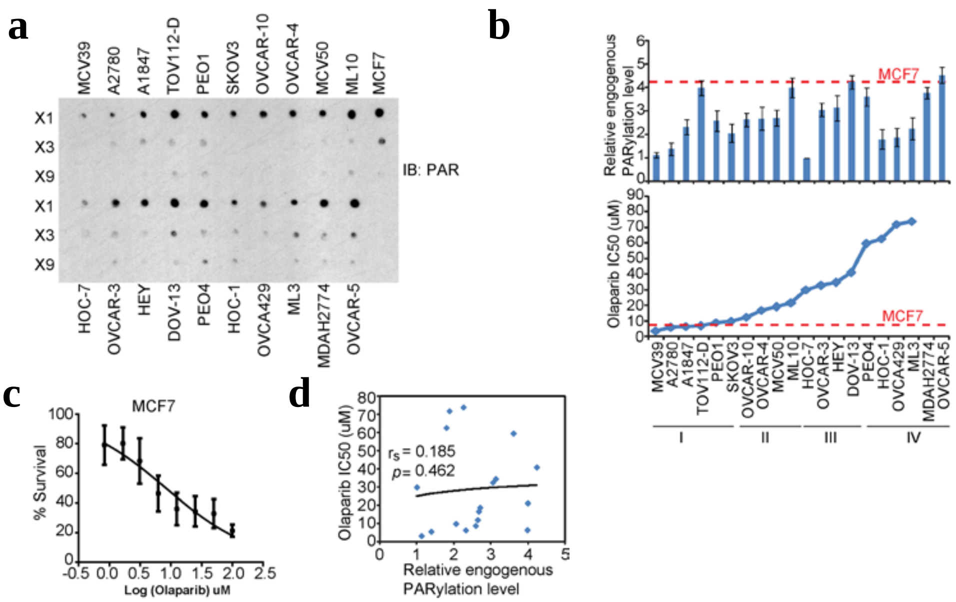

Supplementary Figure 5 Sentivity of ovarian cancer cells to olaparib is not associated with the endogenous PARylation levels without genotoxic treatment in ovarian cancer cell lines. (**a**) The endogenous levels of PARylation without genotoxic treatment were examined by Dot blotting with PAR-binding reagent (MABE 1031, Millipore). The samples were also diluted three times (x3) or 9 times (x9) in the same Dot blotting to avoid sample loading variation. (**b**) MCF7 was treated with olaparib at indicated doses. The sensitivity of MCF7 to olaparib was assessed by MTT assays. The data were summarized from three independent experiments. Data were presented as mean±SD. (**c**) Relative endogenous PARylation levels without genotoxic treatment were summarized from the Dot blotting results by using ImageJ software. The endogenous PARylation level in MCF7 cells was marked as a dashed line. IC50 value of olaparib was calculated from the MTT assays. The cellular sensitive of MCF7 to olaparib treatment was marked as a dashed line. Three independent experiments were performed. Data were presented as mean ± SD. (**d**) Spearman's correlation analysis between endogenous

PARylation level without genotoxic treatment and IC50 of olaparib. *p* value of Spearman’s correlation was calculated by R function, cor.test().

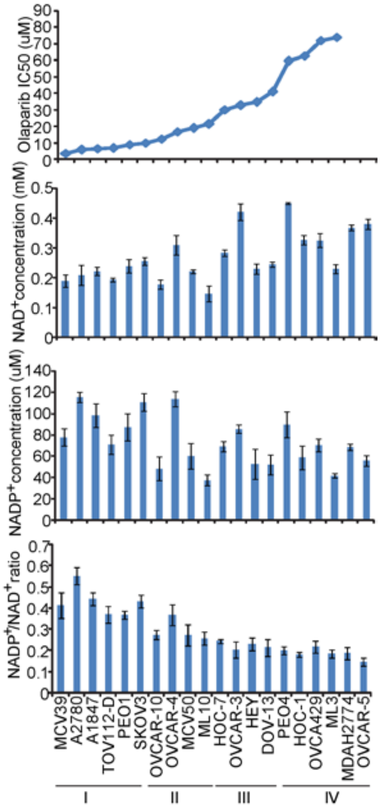

Supplementary Figure 6. **Sentivity of ovarian cancer cells to olaparib is directly associated with the $NADP^+/NAD^+$ ratio.** $NADP^+$ and NAD^+ concentrations were measured in 20 ovarian cancer cell lines and the $NADP^+/NAD^+$ ratio were calculated. $NADP^+/NAD^+$ ratio shows significant correlation with sensitivity of ovarian cancer cells to olarparib. Three independent experiments were performed. Data were presented as mean±SD.

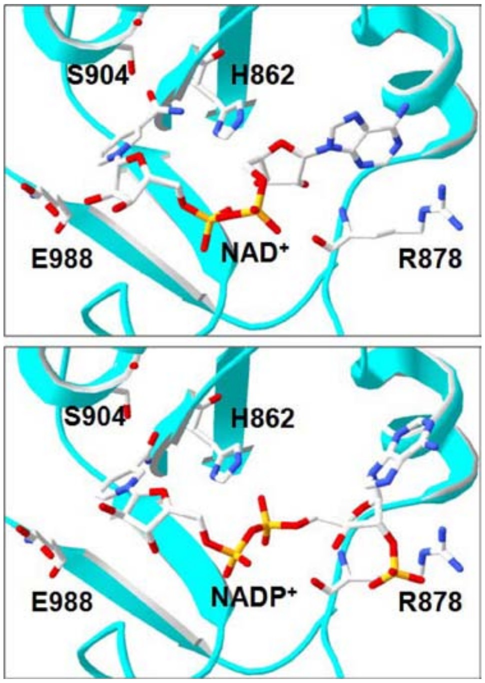

Supplementary Figure 7. The binding mode of NAD^+ and $NADP^+$ in the catalytic pokect of PARP1.

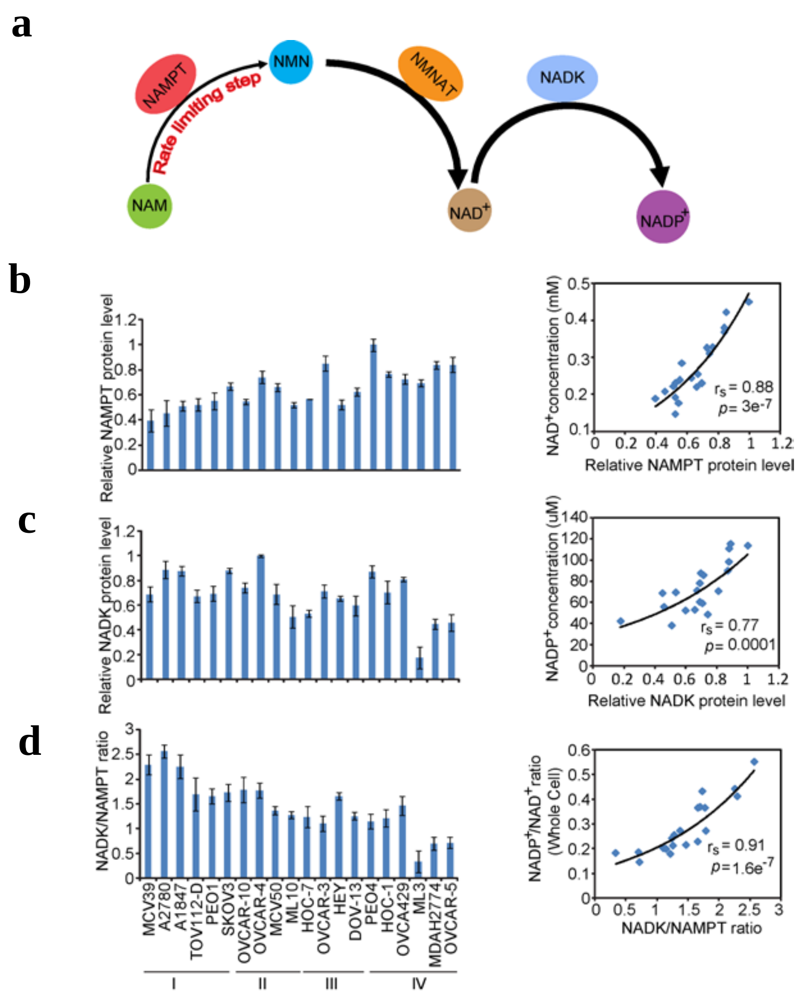

Supplementary Figure 8. Biogenesis of NAD$^+$ and NADP$^+$ plays a key role in determining the NADP$^+$/NAD$^+$ ratio in cells. (**a**) The model of NAD$^+$ and NADP$^+$ biogenesis process. NAMPT is the rate-limiting enzyme during NAD$^+$ biosynthesis. NADK is the solo enzyme converting NAD$^+$ into NADP$^+$. (**b**) Correlation analysis of NAD$^+$ concentrations and NAMPT protein levels.The protein expression level of NAMPT was examined in 20 ovarian cancer cell lines using ELISA. Positive correlation between NAMPT expression level and the NAD$^+$ concentration was observed in the ovarian cancer cells. (**c**) Correlation analysis of NADP$^+$ concentrations and NADK

protein levels.The protein expression level of NADK was examined in twenty ovarian cancer cell lines by ELISA. Positive correlation between NADK expression level and the $NADP^+$ concentration was observed in the ovarian cancer cells. The data were summarized from three independent experiments. Data were presented as mean ± SD. (**d**) Correlation analysis of $NADP^+/NAD^+$ ratio and NADK/NAMPT ratio. The relative ratio of NADK/NAMPT protein expression level was calculated. Positive correlation between NADK/NAMPT ratio and $NADP^+/NAD^+$ ratio was observed. The data were summarized from three independent experiments. Data were presented as mean ± SD. *p* value of Spearman's correlation was calculated by R function, cor.test().

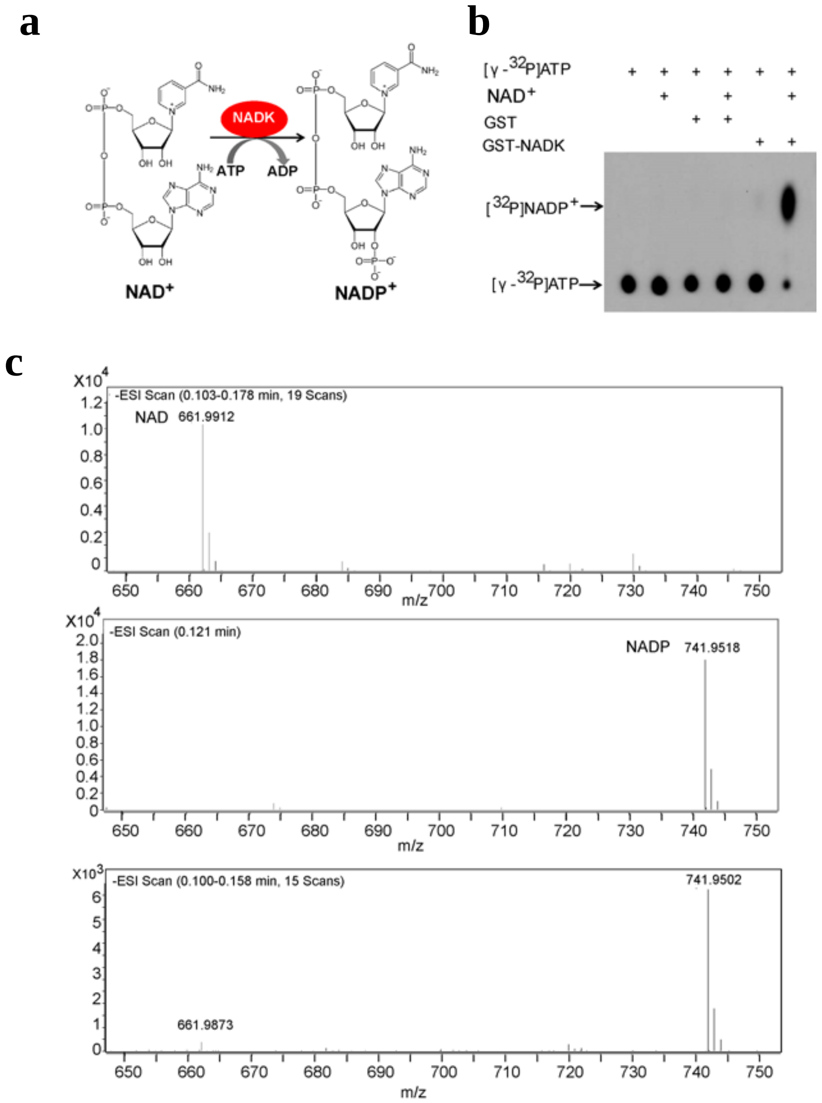

Supplementary Figure 9. NAD$^+$ is converted into NADP$^+$ by NADK. (**a**) A sketch of phosphorylation of NAD$^+$ by NADK. (**b**) TLC analysis shows the kinase activity of recombination GST-NADK, which generats NADP$^+$ by transfering the phosphate moiety from [^{32}P] ATP to NAD$^+$. (**c**) Mass spectrometric analysis shows the conversion of NAD$^+$ into NADP$^+$. The top two panels are standards of NAD$^+$ and NADP$^+$ respectively. The bottom represents the conversion of NAD$^+$ into NADP$^+$ by NADK in vitro.

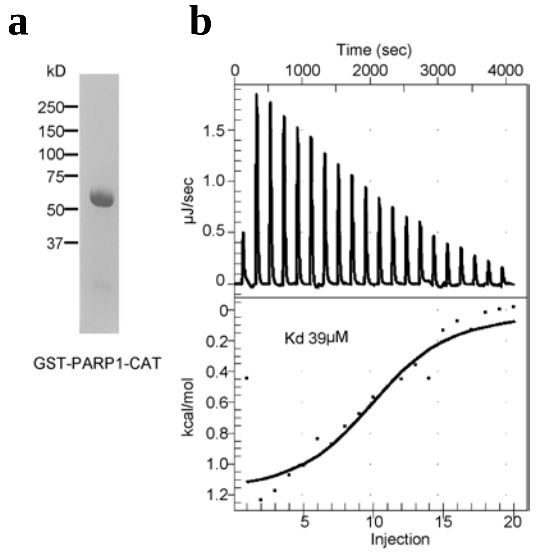

Supplementary Figure 10. The binding affinity between GST-PARP1 CAT and $NADP^+$ was measured by ITC. (**a**) GST-tagged PARP1 catalytic domain (GST-PARP1 CAT, a.a.662-1014) was expressed and purified from *Escherichia coli* BL21. The recombinant protein was analyzed by SDS-PAGE followed by commassie blue staining. (**b**) Titration of $NADP^+$ was injected into a solution containing the purified protein. The inset shows the fit of the data to an equilibrium binding isotherm. The fit provides an equilibrium dissociation constant (Kd) for the binding of $NADP^+$ to the protein.

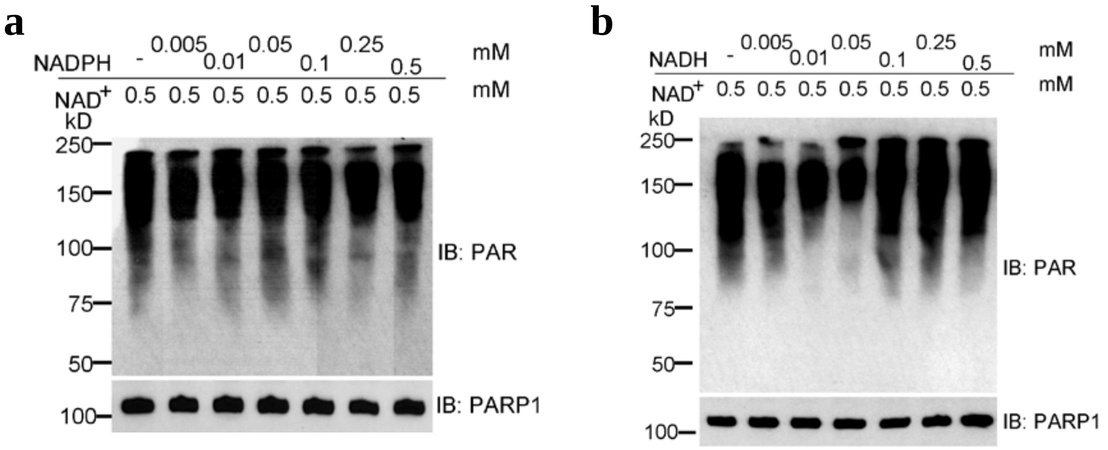

Supplementary Figure 11. Neither NADPH nor NADH supresses PARP1's activity in vitro. In vitro PAPR1-mediated PARylation assay was performed by introducing different ratios of NADPH/NAD$^+$ (**a**) and NADH/NAD$^+$ (**b**). Auto-PARylation of PARP1 was examined by Western blotting with anti-PAR antibody. PARP1 activity was not inhibited by either NADPH or NADH.

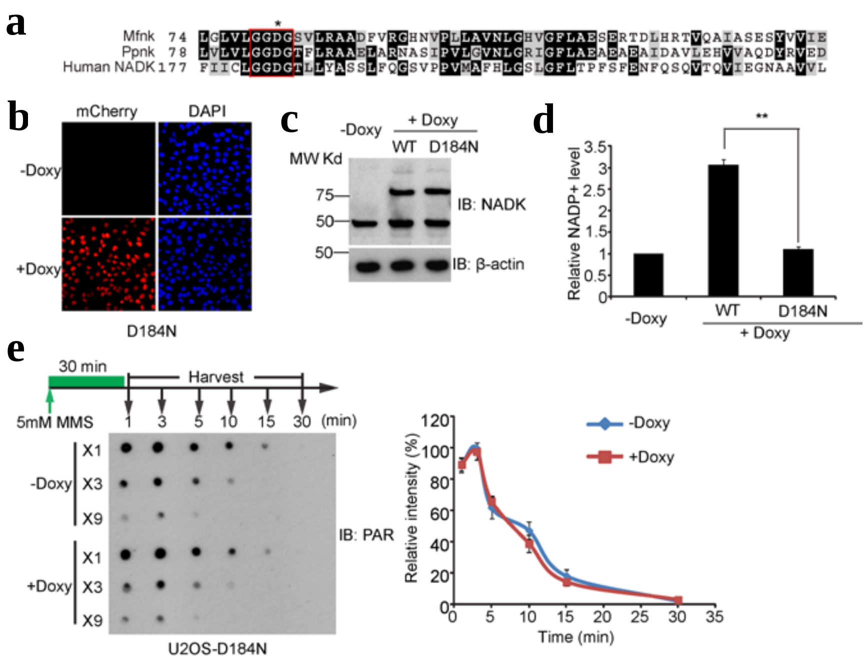

Supplementary Figure 12. Suppression of the DNA damage induced PARylation by NADK depends on its enzymatic activity. (**a**) Multiple alignment of the primary structure of the bacterial NAD(H) kinases and human NADK was performed using ClustalW. The number of residues for each enzyme is specified. The strongly conserved key residues required for the enzymatic activtity are boxed and the D184 reprents the key catalytic residue in human NADK (hNADK). (**b**) Doxycycline induces the expression of the D184N mutant (mCherry) of hNADK in U2OS cells efficiently. (**c**) Ectopic expression of mcherry-fused NADK or to the D184N mutant was examined by Western blotting in U2OS cells with doxycycline induction for 24 hours. β-actin was shown as the loading control. Upper band: mCherry-NADK; lower band: endogenous NADK. (**d**) Relative $NADP^+$ levels were measured in cells with ectopic expression of wild type NADK or the D184N mutant. The D184N mutation abolishes the enzymatic activity of NADK. The data were summarized from three independent experiments. Data were presented as mean ± SD. ** $p < 0.01$. Data were analyzed using two-tailed

unpaired student's *t* tests. (**e**) Ectopic expression of the D184N mutant does not suppress the DNA damage-induced PARylation. The cells were treated with 10 mM MMS. The PARylation levels at indicated time points after DNA damage was examined by Dot blotting with PAR-binding reagent (MABE 1031, Millipore). The samples were also diluted three times (x3) or 9 times (x9) in the same Dot blotting experiment to avoid sample loading variations. Three independent experiments were performed. Data were presented as mean±SD.

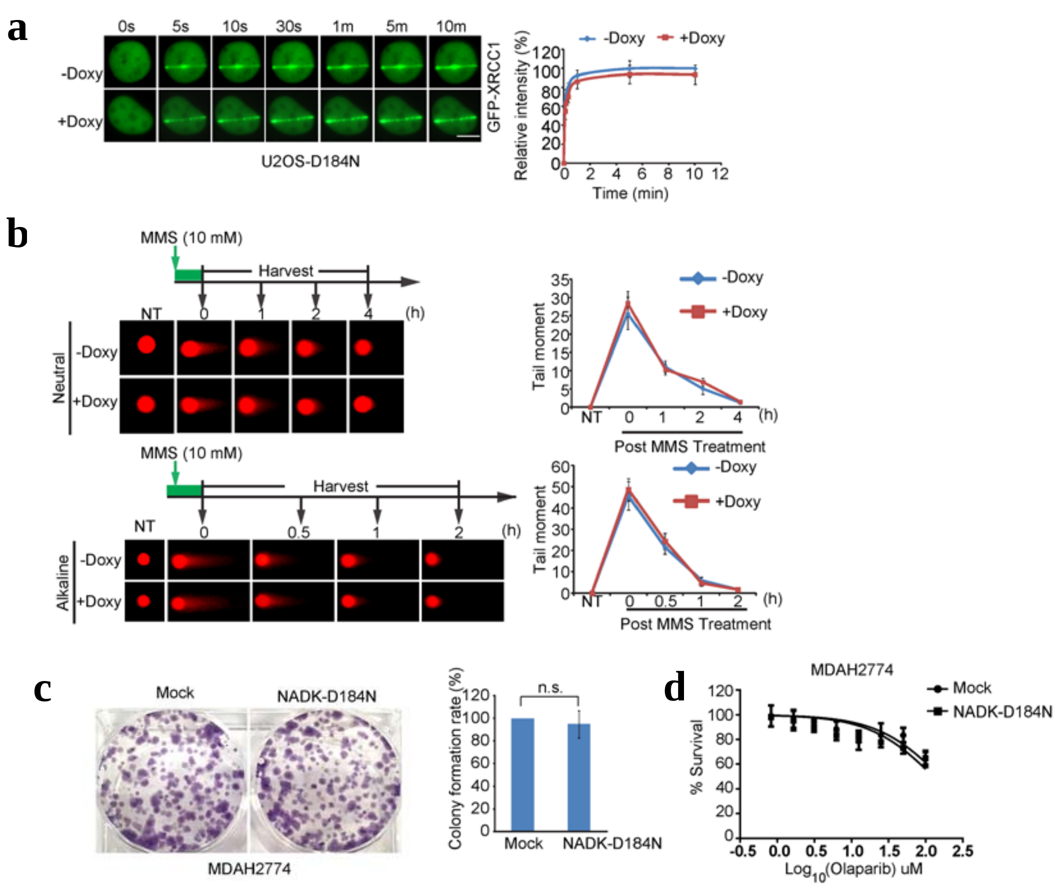

Supplementary Figure 13. Suppression in the PARylation-dependent early DNA damage response by NADK depends on its enzymatic activity.

(**a**) Doxycycline-induced D184N mutant does not suppress the recruitment of the PARylation-dependent DNA damage response. 24 hours after induction, the recruitment kinetics of XRCC1was examined with live cell imaging. The relative intensity on the laser stripes was quantified and summarized in the right panel (mean±SD, from 15 cells in each time point in each experiment). The highest intensity on the laser stripes was calculated as 100 % in each cell, and the kinetics of the recruiment were plotted. Three independent experiments were performed. Bars: 10 μm. (**b**) The D184N mutant do not

affect DNA damage repair. The cells were treated with MMS (10 mM, 30 min). The kinetics of DSB and SSB repair was examined by neutral and alkaline comet assays respectively. Representative comet tails at different time points were shown. The tail moments were summarized from at least 50 cells at each time point in each experiment, and three independent experiments were performed. NT, non-treated. (**c**) The D184N mutant doesn't suppress tumor cell growth. MDAH2774 with exogenous expression of the D184N mutant was examined for colony formation assays. After treatment with 2 μM olaparib for two weeks, the colonies were stained with crystal violet and counted. The results were summarized from three independent experiments. Data were presented as mean ± SD. n.s. not significant. (**d**) Ectopic expression of the D184N mutant does not sensitize tumor cells to olaparib. MDAH2774 with or without exogenous expression of NADK D184N were treated with olaparib at indicated concentrations. MTT assays were performed to examine cell viability. The data were summarized from three independent experiments. Data were presented as mean ± SD.

a

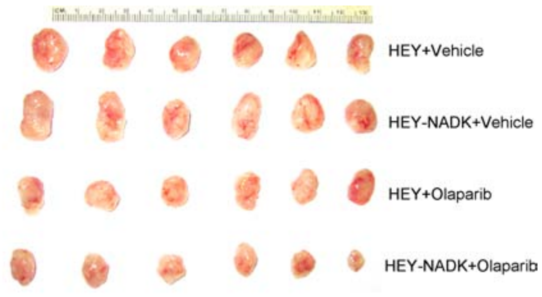

b

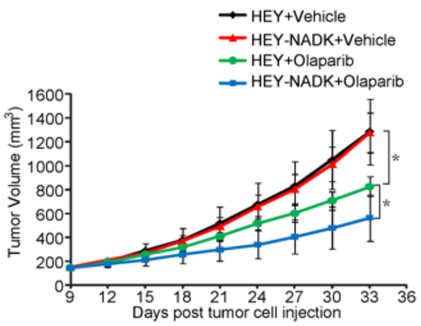

Supplementary Figure 14. High level of NADP$^+$ sensitizes xenograft tumors to olaparib. Ovarian cancer xenografts were initiated by subcutaneous inoculation of HEY cells with or without exogenous expression of NADK in the lower flank of NOD Scid mice as described previously. 5 mg/kg olaparib was given i.p. daily. Controls were dosed with vehicle only. Both digital photograph (**a**) and tumor growth curve (**b**) shows that exogenous expression of NADK significantly sensitized the HEY xenograft tumors to Olaparib treatment. Data were presented as mean±SD. * $p < 0.05$. Data were analyzed using two-tailed unpaired student's *t* tests.

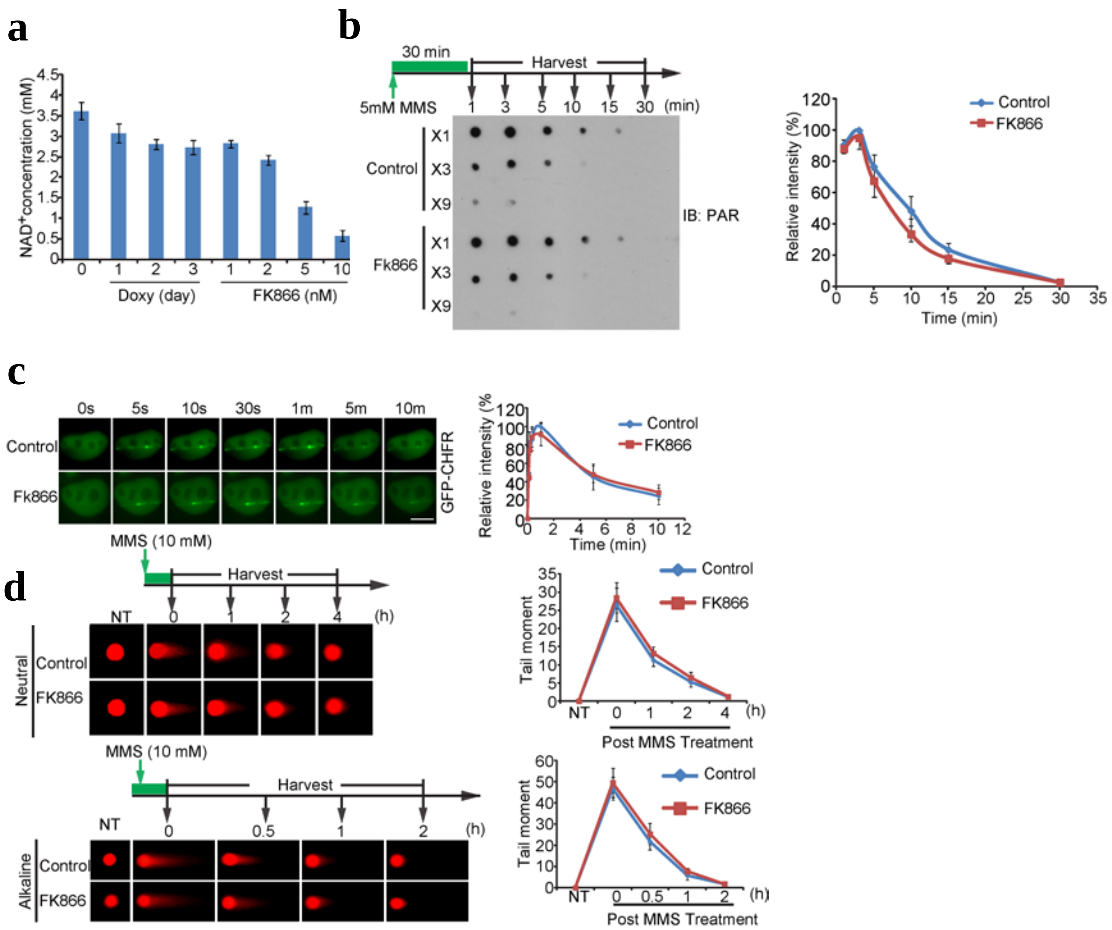

Supplementary Figure 15. The impact of the reduction of NAD$^+$ level on PARylation. (**a**) Relative NAD+ levels in TET-ON NADK U2OS cells with doxycycine and FK866 treatment. Around 25 % decrease in the levels of NAD$^+$ level was caused by either doxycycline or 2 nM FK866 treatment for 72 hours. The data were summarized from three independent experiments. Data were presented as mean ± SD. (**b**) 2 nM FK866 treatment does not abolish the DNA damage-induced PARylation. The cells were treated with 2 nM FK866 for 72 hours followed by 10 mM MMS treatment. Dot blotting was performed to examine PARylation at indicated time points with PAR-binding reagent (MABE 1031, Millipore). The samples were also diluted three times (x3) or 9 times (x9)

in the same Dot blotting experiment to avoid sample loading variations. Three independent experiments were performed. Data were presented as mean±SD. (**c**) FK866 treatment does not suppress the recruitment of the PARylation-dependent DNA damage response factor CHRF. After U2OS cells were treated with 2 nM FK866 for 72 hours, the recruitment kinetics of CHFR was examined with live cell imaging. The relative intensity on the laser stripes were quantified and summarized at the right panel (mean±SD, from 15 cells in each time point in each experiment). The highest intensity on the laser stripes was calculated as 100 % in each cell, and the kinetics of the recruiment were plotted. Three independent experiments were performed. Bars: 10 μm. (**d**) FK866 treatment does not significantly affect DNA damage repair. U2OS cells were treated with FK866 (2 nM, 72 hours) followed by MMS treatment (10 mM, 30 min). The kinetics of DSB and SSB repair was examined by neutral and alkaline comet assays respectively. Representative comet tails at different time points were shown. The tail moments were summarized from at least 50 cells at each time point in each experiment, and three independent experiments were performed. NT, non-treated.

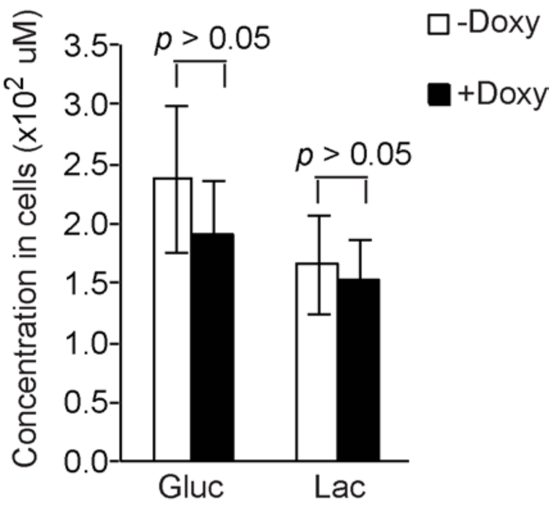

Supplementary Figure 16. The exogenous expression of NADK does not affect glucose metabolism. The glucose metabolism in U2OS cells was examined by Bioanalyzer. which included glucose uptake and lactate production. NADK overexpression induced by doxycyline doesn’t change glucose metabolism significantly in U2OS cells. Data were analyzed using two-tailed unpaired student’s *t* tests.

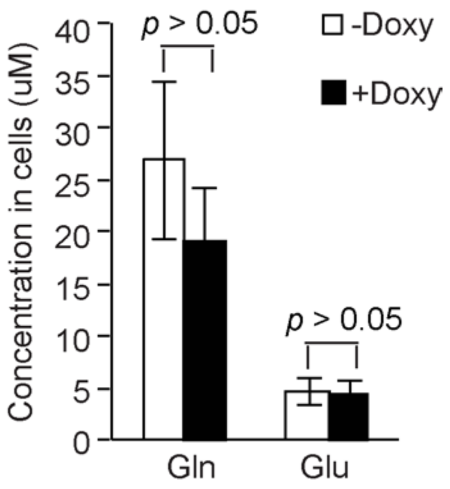

Supplementary Figure 17. The exogenous expression of NADK does not affect glutamine metabolism. The glutamine metabolism in U2OS cells was examined by Bioanalyzer. which included the glutamine uptake and glutamate production. NADK overexpression induced by doxycyline doesn't change glutamine metabolism significantly in U2OS cells. Data were analyzed using two-tailed unpaired student's *t* tests.

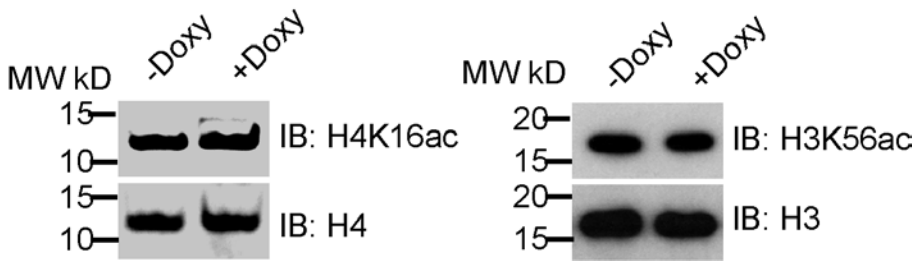

Supplementary Figure 18. The exogenous expression of NADK does not affect deacetylation substrates of Sirt1. Histone H4K16 and H3K56 acetylation were examined by Western blotting in U2OS cells with or without doxycycline treatment. Histone H4 and H3 blotting were shown as loading control respectively.

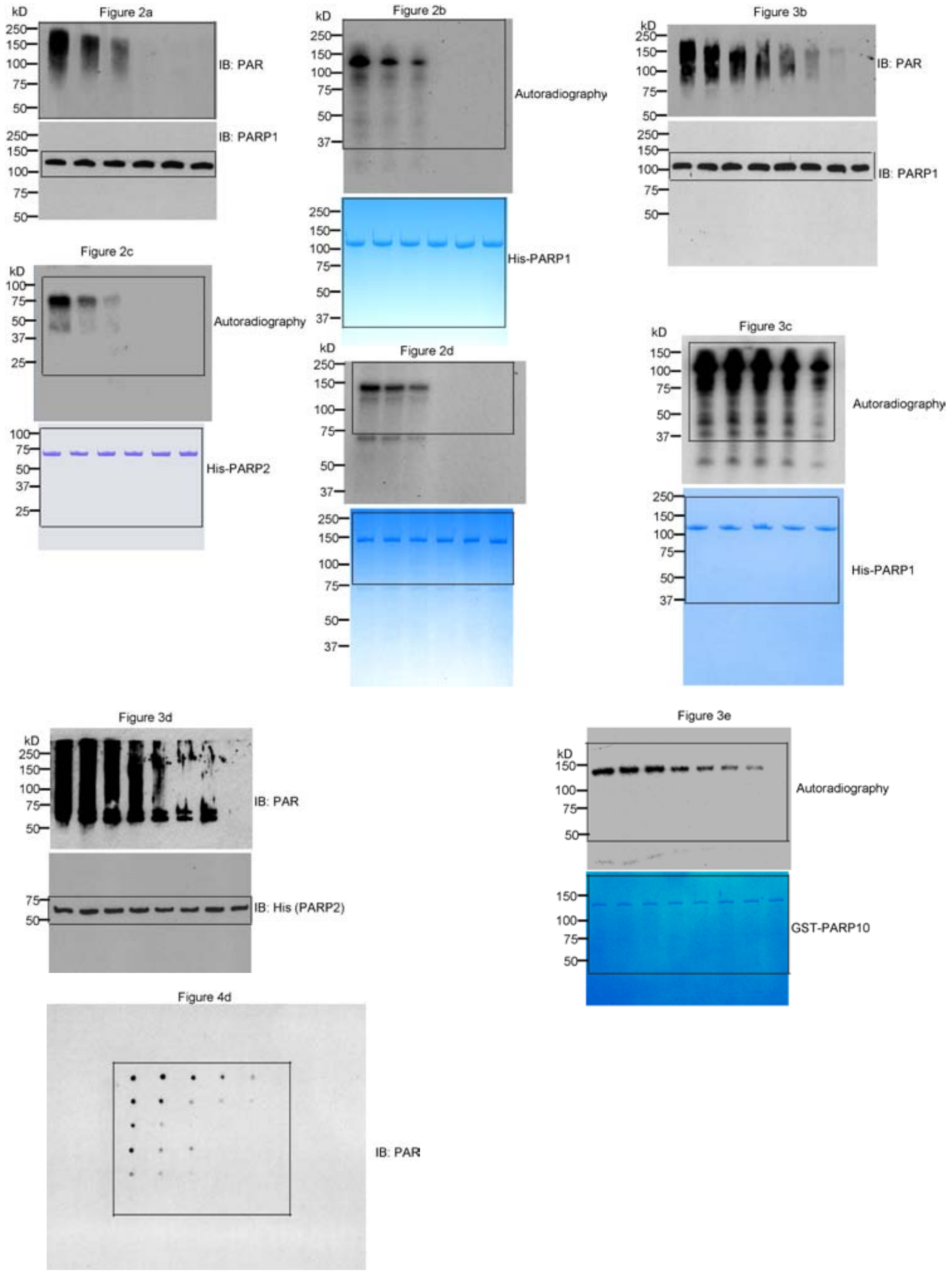

Supplementary Figure 19. The uncropped images of all blots and gels shown in the major figures of this study.
