# Supplementary material for: NADP+ is an endogenous PARP inhibitor in DNA damage response and tumor suppression
Source: Nat Commun. 2019 Feb 11;10:693. doi: 10.1038/s41467-019-08530-5 (PMC6370829; doi:10.1038/s41467-019-08530-5)
Supplement: Supplementary file 2 — Reporting Summary [file 41467_2019_8530_MOESM2_ESM.pdf]

nature research

Corresponding author(s): Xiaochun Yu

Last updated by author(s): Dec 11, 2018

Reporting Summary

Nature Research wishes to improve the reproducibility of the work that we publish. This form provides structure for consistency and transparency in reporting. For further information on Nature Research policies, see Authors & Referees and the Editorial Policy Checklist.

Statistics

For all statistical analyses, confirm that the following items are present in the figure legend, table legend, main text, or Methods section.

n/a	Confirmed	
☐	☒	The exact sample size (*n*) for each experimental group/condition, given as a discrete number and unit of measurement
☐	☒	A statement on whether measurements were taken from distinct samples or whether the same sample was measured repeatedly
☐	☒	The statistical test(s) used AND whether they are one- or two-sided<br>*Only common tests should be described solely by name; describe more complex techniques in the Methods section.*
☐	☒	A description of all covariates tested
☐	☒	A description of any assumptions or corrections, such as tests of normality and adjustment for multiple comparisons
☐	☒	A full description of the statistical parameters including central tendency (e.g. means) or other basic estimates (e.g. regression coefficient) AND variation (e.g. standard deviation) or associated estimates of uncertainty (e.g. confidence intervals)
☒	☐	For null hypothesis testing, the test statistic (e.g. *F*, *t*, *r*) with confidence intervals, effect sizes, degrees of freedom and *P* value noted<br>*Give P values as exact values whenever suitable.*
☒	☐	For Bayesian analysis, information on the choice of priors and Markov chain Monte Carlo settings
☒	☐	For hierarchical and complex designs, identification of the appropriate level for tests and full reporting of outcomes
☐	☒	Estimates of effect sizes (e.g. Cohen's *d*, Pearson's *r*), indicating how they were calculated

Our web collection on statistics for biologists contains articles on many of the points above.

Software and code

Policy information about availability of computer code

Data collection	FACSdiva was used to collect the flow cytometry data.
Data analysis	Spearman’s correlation was performed using the online software, Wessa, P. (2017), (Free Statistics Software, Office for Research Development and Education, version 1.1.23-r7, URL http://www.wessa.net/). FlowJo was used to analyze the flow cytometry data. Gray values for Western blotting and Dot blotting results were analyzed using ImageJ.

For manuscripts utilizing custom algorithms or software that are central to the research but not yet described in published literature, software must be made available to editors/reviewers. We strongly encourage code deposition in a community repository (e.g. GitHub). See the Nature Research guidelines for submitting code & software for further information.

Data

Policy information about availability of data

All manuscripts must include a data availability statement. This statement should provide the following information, where applicable:

- Accession codes, unique identifiers, or web links for publicly available datasets
- A list of figures that have associated raw data
- A description of any restrictions on data availability

Uncropped blots and gels of major figures are shown in Supplementary Figure 19 and all data are available from the corresponding author upon reasonable request.

Field-specific reporting

Please select the one below that is the best fit for your research. If you are not sure, read the appropriate sections before making your selection.

☒ Life sciences ☐ Behavioural & social sciences ☐ Ecological, evolutionary & environmental sciences

For a reference copy of the document with all sections, see nature.com/documents/nr-reporting-summary-flat.pdf

Life sciences study design

All studies must disclose on these points even when the disclosure is negative.

Sample size	6 mice per group were used in the mouse xenograft models study.
Data exclusions	In the mouse xenograft models study, only tumors with diameter of > 0.3 cm were considered.
Replication	Experiments were repeated so that our data are based on at least two or three independent experiments with similar results. The precise number of repeats are given in the figure legend.
Randomization	We did not use randomization to assign animals to experimental groups.
Blinding	The data presented did not require the use of blinding.

Reporting for specific materials, systems and methods

We require information from authors about some types of materials, experimental systems and methods used in many studies. Here, indicate whether each material, system or method listed is relevant to your study. If you are not sure if a list item applies to your research, read the appropriate section before selecting a response.

Materials & experimental systems

n/a	Involved in the study	
☐	☒	Antibodies
☐	☒	Eukaryotic cell lines
☒	☐	Palaeontology
☐	☒	Animals and other organisms
☒	☐	Human research participants
☒	☐	Clinical data

Methods

n/a	Involved in the study	
☒	☐	ChIP-seq
☐	☒	Flow cytometry
☒	☐	MRI-based neuroimaging

Antibodies

Antibodies used	Rabbit anti-NADK antibody (#15548-1-AP, 1:2000 dilution) was purchased from Proteintech Group, Inc.<br>Mouse anti-β-actin monoclonal antibody (AC-15) (#A1978, 1:10000 dilution) was purchased from Sigma.<br>Mouse anti-PAR monoclonal antibody (#4335-MC-100, 1:2000 dilution) was purchased from Trevigen.<br>Anti-poly-ADP-ribose binding reagent (#MABE1031, 1:500 dilution) was purchased from Millipore.<br>Rabbit anti-H3 polyclonal antibody (#06-755, 1:2000 dilution), Rabbit anti-H4 polyclonal antibody (#07-108, 1:2000 dilution), and Rabbit anti-H4K16ac polyclonal antibody (#07-329, 1:2000 dilution) were purchased from Millipore.<br>Rabbit anti-H3K56ac polyclonal antibody (#4243, 1:2000 dilution), Rabbit anti-PARP1 monoclonal antibody (#9532, 1:2000 dilution), and mouse anti-His tag monoclonal antibody (27E8) (#2366, 1:2000 dilution) were purchased from Cell Signaling Technology.
Validation	All antibodies were validated by the supplier for human samples, and were checked in the lab by Western blotting on cell lysate and by comparing to the manufacturer's results.

Eukaryotic cell lines

Policy information about cell lines

Cell line source(s)	Ovarian cancer cell lines used in this study were described in Methods section. U2OS and MCF7 cells were acquired from ATCC.
Authentication	U2OS and MCF7 cell lines were authenticated using STR analysis.
Mycoplasma contamination	All cell lines are frequently tested for mycoplasma contamination. Cell lines used in this study were verified to be

Mycoplasma contamination	mycoplasma negative before undertaking any experiments.
Commonly misidentified lines (See ICLAC register)	No cell lines used are listed in the database of commonly misidentified cell lines.

Animals and other organisms

Policy information about studies involving animals; ARRIVE guidelines recommended for reporting animal research

Laboratory animals	Description of research mice used for experiments can be found in the relevant figure legends and Methods.
Wild animals	No wild animals were used in this study.
Field-collected samples	No field-collected samples were used in this study.
Ethics oversight	All the animal experiments were performed in accordance with National Institute of Health animal use guidelines and protocols after approval by City of Hope Beckman Research Institute Animal Care and Use Committee.

Note that full information on the approval of the study protocol must also be provided in the manuscript.

Flow Cytometry

Plots

Confirm that:

☒ The axis labels state the marker and fluorochrome used (e.g. CD4-FITC).

☒ The axis scales are clearly visible. Include numbers along axes only for bottom left plot of group (a 'group' is an analysis of identical markers).

☒ All plots are contour plots with outliers or pseudocolor plots.

☒ A numerical value for number of cells or percentage (with statistics) is provided.

Methodology

Sample preparation	This information is included in the methods section page.
Instrument	FACS lSR Fortessa (BD biosciences).
Software	FACSDiva software (BD biosciences) and FlowJo software were used to collect and analyze flow cytometry data.
Cell population abundance	Flow cytometry was used for quantification purpose only (i.e. no post-sorting factions were collected).
Gating strategy	FSC-A/SSC-A gates of the starting cell population were used to dicriminate between viable cells and cells deribris. Singlet and doublet cells were discriminated using FSC-A/FSC-W gating. For transgenic cells expressing GFP, parental cells not expressing the GFP protein were used used as negative controls.

☒ Tick this box to confirm that a figure exemplifying the gating strategy is provided in the Supplementary Information.
